# NCResNet: Noncoding Ribonucleic Acid Prediction Based on a Deep Resident Network of Ribonucleic Acid Sequences

**DOI:** 10.3389/fgene.2020.00090

**Published:** 2020-02-28

**Authors:** Sen Yang, Yan Wang, Shuangquan Zhang, Xuemei Hu, Qin Ma, Yuan Tian

**Affiliations:** ^1^ Key Laboratory of Symbol Computation and Knowledge Engineering of Ministry of Education, and College of Computer Science and Technology, Jilin University, Changchun, China; ^2^ School of Artificial Intelligence, Jilin University, Changchun, China; ^3^ Department of Biomedical Informatics, College of Medicine, The Ohio State University, Columbus, OH, United States

**Keywords:** noncoding RNA, protein coding RNA, RNA sequence features, deep neural networks, noncoding RNA prediction

## Abstract

Noncoding RNA (ncRNA) is a kind of RNA that plays an important role in many biological processes, diseases, and cancers, while cannot translate into proteins. With the development of next-generation sequence technology, thousands of novel RNAs with long open reading frames (ORFs, longest ORF length > 303 nt) and short ORFs (longest ORF length ≤ 303 nt) have been discovered in a short time. How to identify ncRNAs more precisely from novel unannotated RNAs is an important step for RNA functional analysis, RNA regulation, *etc*. However, most previous methods only utilize the information of sequence features. Meanwhile, most of them have focused on long-ORF RNA sequences, but not adapted to short-ORF RNA sequences. In this paper, we propose a new reliable method called NCResNet. NCResNet employs 57 hybrid features of four categories as inputs, including sequence, protein, RNA structure, and RNA physicochemical properties, and introduces feature enhancement and deep feature learning policies in a neural net model to adapt to this problem. The experiments on benchmark datasets of 8 species shows NCResNet has higher accuracy and higher Matthews correlation coefficient (MCC) compared with other state-of-the-art methods. Particularly, on four short-ORF RNA sequence datasets, specifically mouse, *Saccharomyces cerevisiae*, zebrafish, and cow, NCResNet achieves greater than 10 and 15% improvements over other state-of-the-art methods in terms of accuracy and MCC. Meanwhile, for long-ORF RNA sequence datasets, NCResNet also has better accuracy and MCC than other state-of-the-art methods on most test datasets. Codes and data are available at https://github.com/abcair/NCResNet.

## Introduction

Non-coding RNA (ncRNA) cannot translate protein, but it is involved in many crucial and essentially biological processes, such as gene expression (Wang et al., 2019), gene regulation (Deaton and Bird, 2011; Dykes and Emanueli, 2017), gene silencing (Singh et al., 2018), etc. Furthermore, ncRNA plays a key role in the development of diverse cancers, including pancreatic cancer (Peng et al., 2016; Xiong et al., 2017), lung cancer (Anastasiadou et al., 2017), and so on. With the rapid development of next-generation sequencing technology, numerous novel transcripts have been discovered. The recognition of ncRNAs from protein-coding RNAs (pcRNAs) is the first and vital step in exploring the latent function of unannotated transcripts. However, the differentiation of ncRNAs from numerous unclassified sequences is time- and labor-consuming with the use of biological experimental methods (Lu et al., 2019). Furthermore, there is growing evidence that some ncRNAs with short open reading frames (longest ORF length ≤ 303 nt) can translate into micro-peptides (Galindo et al., 2007; Kondo et al., 2007; Kondo et al., 2010; Chng et al., 2013; Magny et al., 2013; Pauli et al., 2014; Anderson et al., 2015), which makes it more difficult to distinguish ncRNAs from pcRNAs. From a computational perspective, many methods have been developed to distinguish ncRNAs and pcRNAs based on machine learning techniques. Liu J et al. proposed a classified method, called CONC (Liu et al., 2006), which employs 180 hybrid features of eight categories and is trained by support vector machine (SVM) (Huang et al., 2018). However, the calculation of CONC is slow with big datasets due to the search and alignment of the dataset. To alleviate the problem of inefficiency, coding protein potential (CPC) (Kong et al., 2007) used SVM to appraise RNA noncoding potential by using six biological sequence features. However, the performance of CPC heavily relies on the quality of multiple sequence alignment (McDermaid et al., 2018). Owing to the lower efficiency of alignment and dataset searches, an alignment-free tool, coding-potential assessment tool (CPAT) (Wang et al., 2013), based on the logistic regression method, predicts ncRNAs by four pure sequence features. Additionally, to accelerate the computational speed of CPC, coding potential calculator version 2 (CPC2) (Kang et al., 2017), an updated version of CPC, uses sequence intrinsic features to differentiate ncRNAs from pcRNAs by SVM. Many previous methods aim to categorize long noncoding RNAs (lncRNAs) and pcRNAs such as iSeeRNA (Sun et al., 2013a), Coding-Non-Coding Index (CNCI) (Sun et al., 2013b), PLEK (Li et al., 2014), FEELnc (Wucher et al., 2017), DeepLNC (Tripathi et al., 2016), COME (Hu et al., 2017), LncRNAnet (Baek et al., 2018), and LncFinder (Han et al., 2018). iSeeRNA, CNCI, PLEK, and LncFinder adapt SVM as a classifier. iSeeRNA works with conservation, ORF, and sequence features. CNCI combines profiling adjoining nucleotide triplets and unequal distribution of codons (codon bias) to construct input features. PLEK uses k-mer scheme features to represent a sequence. LncFinder introduces sequential, RNA secondary structural, and physicochemical properties to build input features. FFFLnc and COME apply the random forest algorithm as a classifier. FFFLnc accepts ORF coverage, codon usage, and multi k-mer frequencies as encoding features. COME utilizes experimental and sequence-based features to assemble the input feature. LncRNANet and DeepLNC manipulate deep neural networks as a predictor. LncRNANet receives a raw RNA sequence, ORF length, and ORF coverage features to learn recurrent neural networks (De Mulder et al., 2015) and convolutional neural networks (Rawat and Wang, 2017). DeepLNC uses multi k-mer frequencies as features to train a deep neural network.

There is growing evidence that some ncRNAs contain short ORFs that can encode small molecule peptides. This discovery illustrates that the categorization of ncRNAs and pcRNAs is more challenging than before. Current computational methods, including all of the methods mentioned above, have yielded encouraging results in distinguishing RNA sequences with long ORFs but do not adapt to distinguishing RNA sequences with short ORFs. To improve the predicted accuracy on short-ORF RNA sequences, CPPred (Tong and Liu, 2019) utilizes composition, transition and distribution (CTD) features (Dubchak et al., 1995), sequence features, and protein features to identify ncRNA by the SVM model. However, the generation of CPPred on cross-species datasets is worse. Therefore, more contributing features and a more powerful classification model are needed to solve the problem.

In this paper, we propose a novel deep learning model, named NCResNet, to identify noncoding RNA. NCResNet combines 57 reasonable features and a modified deep residual network (He et al., 2016) to find ncRNAs. The 57 reasonable features are selected from the sequence, protein, RNA structure, and RNA physicochemical properties to overcome the shortcoming that single or a few types of features cannot represent a raw sequence abundantly and amply. NCResNet modified residual network is a deep learning-based model composed of four main modules: an input module, a feature enhancement module, a deep feature learning module, and a prediction module. Based on feature enhancements and deep feature learning policies, NCResNet achieves better performance than other state-of-the-art methods, such as CPC2, CPAT, IRSOM, LncFinder, and CPPred. On eight benchmark datasets, NCResNet successfully identifies ncRNAs from pcRNAs. In particular, on short-ORF RNA sequence datasets of mouse, *Saccharomyces cerevisiae*, zebrafish, fruit fly, and cow species, NCResNet achieves more than 10 and 15% improvement over the compared methods in terms of accuracy and MCC, respectively. In addition, for long-ORF RNA sequence datasets, NCResNet performs better than other methods on most test datasets. Overall, NCResNet is a robust and high confidence tool for distinguishing ncRNAs and pcRNAs, especially, in short-ORF RNA sequences.

## Methods

### Data

NCRestNet is trained on three-fourths of a human dataset and tested on the rest of the human datas*et al*ong with other seven cross-species datasets of mouse, *S. cerevisiae*, zebrafish, fruit fly, cow, rat, and *Caenorhabditis elegans*. The whole human dataset consists of 33,045 ncRNAs and 42,242 pcRNAs collected from CPPred research. The test datasets of mouse, *S. cerevisiae*, zebrafish, and fruit fly are also derived from CPPred research, containing 20,776/20,776, 826/826, 11,049/11,049, and 4,479/4,479 ncRNAs/pcRNAs, respectively. The other three species test datasets of cow, rat, and *C. elegans* are downloaded from Ensembl (Zerbino et al., 2018) and NONCODE (Bu et al., 2012) including 1,028/1,028, 5,669/5,669, and 2,075/2,075 ncRNAs/pcRNAs, respectively. Moreover, all test datasets of each species are split into long-ORF and short-ORF RNA sequence datasets based on whether the longest ORF length of a sequence is larger than 303 nt. For further verification, we test NCResNet on an independent dataset downloaded from RefLnc (Jiang et al., 2019) research, which contains 20,364 novel long-ORF ncRNAs and 7,142 novel short-ORF ncRNAs assembled from real clinical samples and without overlap of the previous training and test datasets. In this paper, ncRNAs and pcRNAs are treated as negative and positive samples, respectively. **Table 1** shows the number of samples in each species dataset.

**Table 1:** Sample size of each species test dataset.

	Long-ORF RNAs		Short-ORF RNAs	
	NC	PC	NC	PC
Human	8,241	8,241	641	641
Mouse	19,930	19,930	846	846
*S. cerevisiae*	413	413	413	413
Zebrafish	10,662	10,662	387	387
Fruit fly	4,098	4,098	381	381
Cow	284	284	744	744
Rat	4,589	4,589	1,080	1,080
*C. elegans*	582	582	1,493	1,493

**Figure 1:**
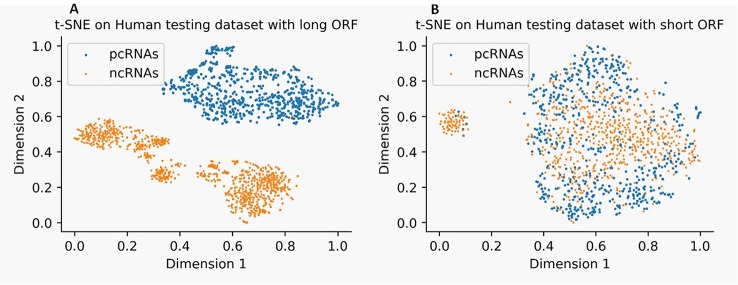
Distribution of noncoding RNA (ncRNAs) and protein-coding RNAs (pcRNAs) on human test datasets with long-open-reading frame (ORF) **(A)** and short-ORF **(B)** RNA sequences by t-Distributed Stochastic Neighbor Embedding (t-SNE).

We use t-Distributed Stochastic Neighbor Embedding (t-SNE) (Gisbrecht et al., 2015) to visualize the distribution of ncRNAs and pcRNAs by mapping 57 features dimensions into two-dimensional space. **Figure 1** illustrates that ncRNAs and pcRNAs with long-ORF sequences in the human test dataset can be easily classified by these features (see **Figure 1A**), while datasets with short-ORF sequences are harder (see **Figure 1B**), which reconfirms the observation that categorization between ncRNAs and pcRNAs is more challenging in short-ORF RNAs.

### Feature Construction

Many ncRNA identification methods have been attempted, and most of them just use features from sequence information alone. However, in this work, we employ 57 hybrid features from four categories: sequence, protein, RNA structure, and RNA physicochemical properties.

#### RNA Sequence Features

There are essential differences between ncRNAs and pcRNAs at the sequence level (Ransohoff et al., 2018). For example, ncRNAs often contain shorter ORFs and lack sequence conservation. In this paper, 16 features generated by sequence are employed. Firstly, the first three features related to ORFs, including ORF length, ORF coverage, and ORF integrity [whether containing open-reading frame (ORF)], are chosen because short-ORF RNAs have a low ability to translate proteins. Secondly, the Fickett score (Fickett, 1982) is a linguistic feature that distinguishes ncRNAs and pcRNAs according to the combinational effect of nucleotide composition and codon usage bias. Hexamer score (Wang et al., 2013) is also an essential feature because of the dependence between adjacent amino acids in proteins. Thirdly, we select some fundamental sequence features such as the codon number, codon ratio, GC content, as well as GC variance. Fourthly, we use the distance between ncRNAs and pcRNAs as candidate features, including Dist.Ratio which is the ratio between Euclidean-distance to ncRNAs and Euclidean-distance to pcRNAs (Han et al., 2018).

#### RNA Structure Features

RNA structure plays significant roles in some biological functions (Burge et al., 2013) and is considered more conserved than the primary sequence but is seldom employed to predict ncRNA. Therefore, we introduce some RNA structure characters as features. Recently, composition, transition, and distribution (CTD) were found to be connected with RNA structure and are seldom used to identify ncRNAs. Therefore, in this paper, we use CTD to represent RNA structure information. CTD includes 30 features from the three categories of composition, transition, and distribution. The composition features are the number of amino acids of a particular property divided by the total number of amino acids; transition features characterize the percent frequency with which amino acids of a particular property are followed by amino acids of a different property; and the distribution features to measure the chain length within which the first, 25, 50, 75, and 100% of the amino acids of a particular property are located.

For example, we use a toy RNA sequence ATACGTACTGCTGACGTAGC which contains five adenines (As), five thymines (Ts), five guanines (Gs), and five cytidines (Cs) to show how to calculate the CTD features. The composition includes four features which are frequency of adenines, thymines, guanines, and cytidines respectively. The toy RNA sequence contains 5 A, 5 T, 5 G, and 5 C, so composition is equal to 5/20 = 0.25, 5/20 = 0.25, 5/20 = 0.25, and 5/20 = 0.25. Transition includes AT, AC, AG, TG, TC, and GC six features which describe the percent frequency with the conversion of four nucleotides between adjacent positions. AT represents the percent frequency of A adjoining T or T adjoining A. AC, AG, TG, TC, and GC are the same formulation of AT. Therefore, the transition for the toy RNA sequence is equal to 2/19 = 0.105, 3/19 = 0.158, 2/19 = 0.105, 4/19 = 0.211, 2/19 = 0.105, 4/19 = 0.211. Distribution is five relative positions along the transcript sequence of each nucleotide, with 0 (first node), 25, 50, 75, 100% (last node), to measure the nucleotide distribution. For As, the 0% is location at first position in toy RNA sequence, 25, 50, 75, and 100% at 3rd, 7th, 14th, 18th position respectively. So, As are 1/20 = 0.05, 3/20 = 0.15, 7/20 = 0.35, 14/20 = 0.7, and 18/20 = 0.9. Likewise, Ts, Gs, and Cs are 0.1, 0.3, 0.45, 0.6, 0.85, 0.25, 0.5, 0.65, 0.8, 0.95, 0.2, 0.4, 0.55, 0.75, 1. We use A0, A1, A2, A3, A4, T0, T1, T2, T3, T4, G0, G1, G2, G3, G4, C0, C1, C2, C3 and C4 to represent the 20 features.

#### Protein Features

ncRNAs cannot translate proteins, so the fake protein sequence translated by ncRNA does not have true protein sequence characters. Based on this understanding, we select and calculate six related protein characters as features, including protein instability index, grand average of hydropathy (GRAVY), isoelectric point, molecular weight and their combination by Biopython (Cock et al., 2009).

#### Physicochemical Property Features

pcRNA has a different power spectrum distribution compared with ncRNAs (Han et al., 2018). Generally, in the power spectrum of a protein-coding transcript, a peak value will emerge in the thirds position but will not appear in ncRNA (Han et al., 2018). For any DNA sequence, nucleotides can be replaced by EIIP values: {*A*→0.1260; *C*→0.1340; *G*→0.0806; *T*→0.1335} (Nair and Sreenadhan, 2006). A sequence power spectrum calculated by the following equation:

$$S_{e}[k]={\left|\sum_{n=0}^{N-1}X_{e}[n]e^{-j\frac{2\pi kn}{N}}\right|}^{2}\;(k=0,1,2,\cdots ,N-1)$$

where *X_e_*[*n*] is EIIP indicator value at nth position of a sequence, N is the sequence length.

Based on the difference, we employ six physicochemical properties from power spectrum as features, including Signal.Peak, signal-to-noise ratio (SNR), Signal.Min, Signal.Q1, Signal.Q2, and Signal.Max. Signal.Peak records the third position value (peak value), and SNR is equal to Signal.Peak divided by the averaging power of a sequence. Additionally, the power spectrum of a sequence is sorted in descending order to sample four position values, which are Signal.Min, Signal.Q1, Signal.Q2, and Signal.Max, corresponding to the minimum, lower quartile, upper quartile, and maximum value of sorted power spectrum, respectively.

**Figure 2:**
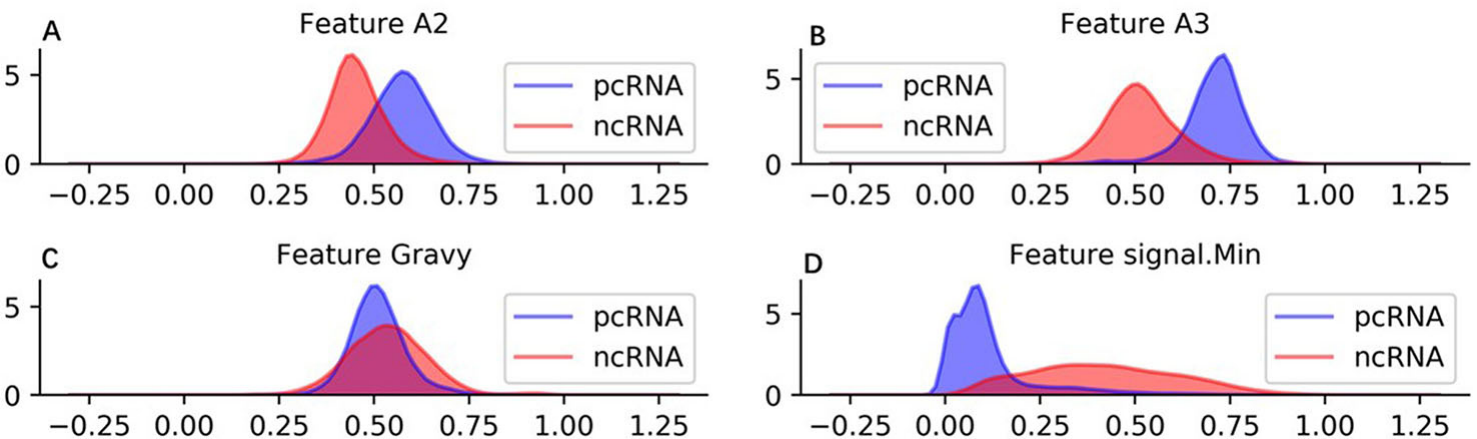
Density distribution of four used features on human training dataset. **(A)** Feature A2 is the rate between location of 25% A and sequence length. **(B)** Feature A3 is the rate between location of 50% A and sequence length. **(C)** Feature Gravy is grand average of hydropathy of a protein. **(D)** signal.Min is minimum power value in sorted power spectrum of a RNA sequence.

In brief, sequence, protein, RNA structure, and physicochemical property information are employed to generate 57 features. These features can represent a raw sequence abundantly and copiously from diverse perspectives. To visual the features intuitively, we show density distribution of four used features on human training ncRNA and pcRNA dataset in **Figure 2**. Additionally, the detail definition and description of all features are listed in **Supplementary Table 1** and the density distribution of all the features on the human training dataset is shown in **Supplementary Table 2**.

### Model Structure

**Figure 3:**
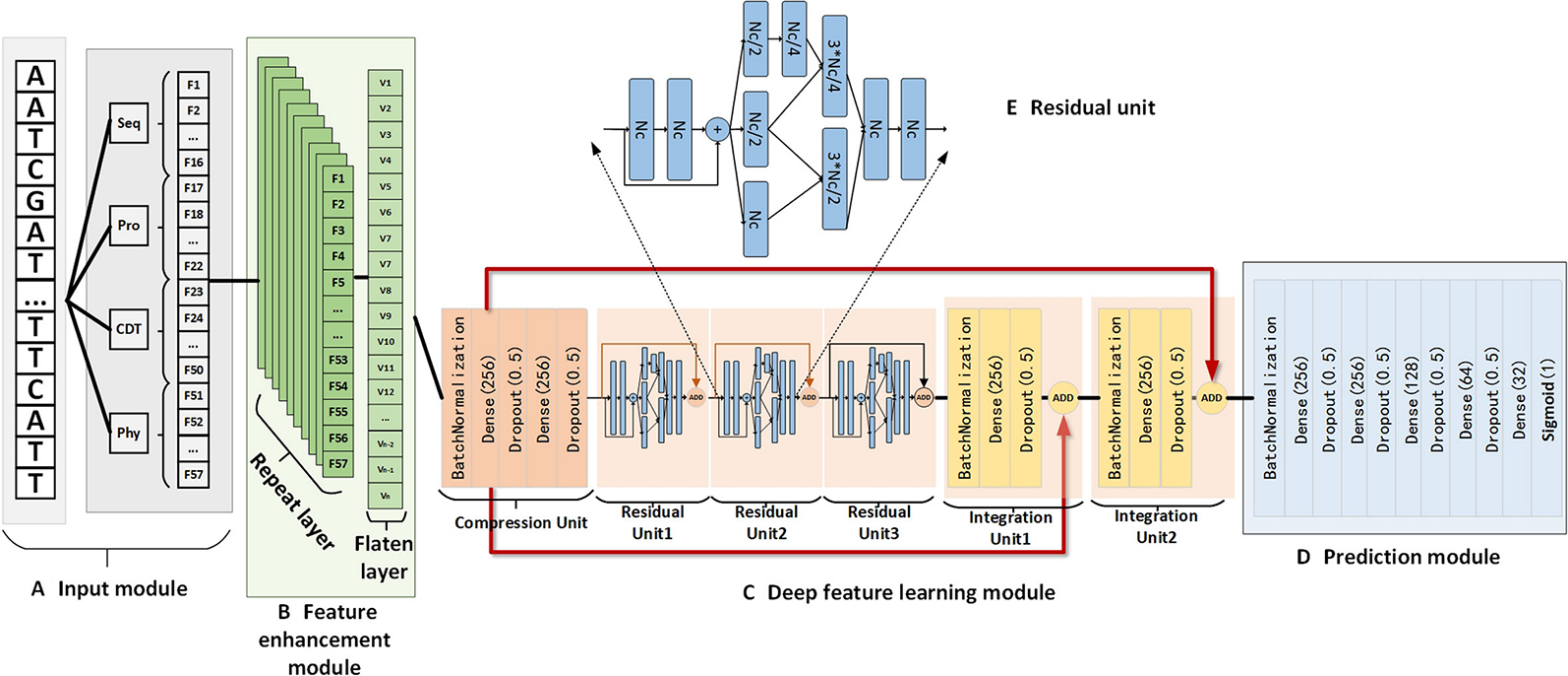
The detailed structure of NCResNet includes the input module **(A)**, feature enhancement module **(B)**, deep feature learning module **(C)**, and prediction module **(D)**. In addition, the deep feature learning module contains multiple residual units which consist of multiple fully connected neural network layers, and the structure of the residual unit is shown in panel **(E)**. Seq, Pro, CTD, and Phy represent sequence features, protein features, CTD features, and RNA physicochemical property features, respectively. *Dt*, *Nc*, *Nr*, and *Pd* are the hyperparameters. *Dt* represents the duplicated time of input features in the feature enhancement module; *Nr* equals the number of residual units *Nc* denotes the cell number of the fully connected neural network layer; *Pd* is the dropout value.

In this paper, we propose a novel deep-learning-based model, named NCResNet, for predicting whether an RNA sequence is an ncRNA or a pcRNA. To achieve the aim, as shown in **Figure 3**, NCResNet is designed to contain four modules, including an input module, feature enhancement module, deep feature learning module, and prediction module. The input module is used to receive an RNA sequence and calculate 57 features related to an RNA sequence, RNA structure, protein features, and RNA physicochemical properties as mentioned above.

The feature enhancement module is designed to enhance feature information represented by 57 features and contains a repeated layer and a flatten layer. The repeated layer is used to duplicate features multiple times to augment the information of the features, where the duplication time is a hyperparameter determined by 10-fold cross validation. Then, the flatten layer is applied to flatten the duplicated features into a linear vector, which can be fed into the deep feature learning module as input.

The deep feature learning module is composed of six basic units, and each unit contains several fully connected neural network layers embedded by dropout layers and a rectified linear unit (ReLU) activation function, where the dropout layer aims to prevent the overfitting of the training model. Meanwhile, a batch normalization operation is employed by the first layers of each unit to guarantee the data derived from different sources follow the same distribution. The principal part of the deep feature learning module has three residual units, which aim to extract the high-level features for an RNA sequence by modified Inception-Resnet (Kamilaris and Prenafeta-Boldú, 2018). Inception-ResNet is a significant deep learning network, which combines the residual and inception methods to relieve gradient vanishing and gradient explosion problems. Based on these strategies, it is feasible to extend the neural network layer deeper and gain deep level features. However, unlike the traditional Inception-ResNet, we combine fully connected neural network layers (Parvat et al., 2017) to construct a residual unit rather than a neural network convolution kernel (Rawat and Wang, 2017). **Figure 3E** shows the structure of the residual unit used in our method. In front of the three residual units, a compression unit, constructed by two fully connected neural network layers, is applied to reduce the dimension of enhanced features to decrease the number of parameters of NCResNet. Behind the three residual units, we assign two integration units made up of a fully connected neural network layer to fuse the advanced features from the front three residual units and the primary features generated by the compression unit. This is an efficient way to supplement the information that is missing in the extraction process for high-level features, according to recent advances in deep learning (Iandola et al., 2014).

The prediction module, stacked by five fully-connected neural network layers, is the last module of NCResNet aimed to receive integrated features from the deep feature learning module to predict ncRNA probability for an RNA sequence. Like the architecture of the units in the deep feature learning module, each fully connected neural network layer is mediated by a dropout operation (Dahl et al., 2013) and a ReLU activation function. Meanwhile, the first fully connected neural network layer applies a batch normalization operation (Ioffe and Szegedy, 2015).

NCResNet is implemented in Python3 using Keras (Parvat et al., 2017) and Tensorflow (Parvat et al., 2017). Additionally, we use the high-performance NVIDIA GeForce GTX2080Ti GPU to accelerate the computation. Binary cross entropy (BCE) (Zhang and Sabuncu, 2018) is applied as the loss function. BCE defined as follows:

$$BCE(p_{i})=y_{i}\log(p_{i})+(1-p_{i})\log(1-p_{i})$$

where *p_i_* is the predicted probability of an ncRNA sequence, and *y_i_* is the label indicating whether it is an ncRNA. NCResNet introduces the AdaBound optimizer to minimize loss function and update parameters in backpropagation. AdaBound is an adaptive optimizer proposed recently, which can achieve the same performance as SGD and as fast as AdaGrad, RMSprop, and Adam (Gambella et al., 2019).

### Performance Evaluation of NCResNet

NCResNet is evaluated by the widely used standard performance metrics, which are sensitivity (SN), specificity (SP), accuracy (ACC), F1-score, and Matthews correlation coefficient (MCC). These evaluation metrics are defined as follows:

$$Sensitivity\;(SN)=\frac{TP}{TP+FN}$$

$$Specificity\;(SP)=\frac{TN}{TN+FP}$$

$$F1-score=\frac{2TP}{2TP+FP+FN}$$

$$Accuracy\;(ACC)=\frac{TP+TN}{TP+TN+FP+FN}$$

$$\begin{array}{l} Matthews\;Correlation\;coefficient\;(MCC)\\ =\frac{TP*TN-FP*FN}{\sqrt{\left(TP+FN)*(TP+FP)*(TN+FP)*(TN+FN\right)}} \end{array}$$

where TP, FP, TN, and FN represent the true positives, false positives, true negatives, and false negatives, respectively. We also plot the receiver operating characteristic curves (ROC) (Hanley and McNeil, 1982) and computed area under the curve (AUC) (Faraggi and Reiser, 2002) to show the distinctly different performance of each predictor.

## Results

### The Effects of Feature Combination

To explore the performance of different combinations of four feature categories, a 10-fold cross validation experiment is conducted on human training datasets. As shown in **Table 2**, on training and test datasets, NCResNet trained with all feature categories achieves better performance and obtains the lowest accuracy when it only uses sequence-based features. The result shows that the integration of four feature categories is a compelling combination for distinguishing ncRNA from pcRNA.

**Table 2:** Effects of feature information in terms of prediction accuracy.

	Seq	Seq, Pro	Seq, Pro, Str	Seq, Pro, Str, Phy
Training	0.8229	0.9634	0.9862	**0.9868**
Test	0.8197	0.9514	0.9707	**0.9828**

Seq, sequence features; Pro, protein features; Str, RNA structure features; Phy, physicochemical property features.

### Hyperparameters of NCResNet Determined by 10 Cross-Validation

The optimal hyperparameters of NCResNet are empirically chosen *via* grid research with a 10-fold cross validation in terms of average accuracy on human training datasets. The baseline hyperparameters are duplication time (*D_t_*) in the features enhancement module, the number of residual units (*N_r_*) in the deep feature learning module, and the number of cells (*N_c_*) of the fully connected neural network layer in the deep feature learning module and dropout probability (*P_d_*), which are 10, 3, 512, and 0.3, respectively. The results of the 10-cross validation experiment are shown in **Table 3**.

**Table 3:** Effects of hyperparameter variations through 10-fold cross validation in terms of prediction accuracy.

Parameters	*D_t_*				*N_r_*				*N_c_*				*P_d_*				
	1	5	10	20	1	3	5	10	64	128	256	512	0	0.3	0.5	0.7
Training	0.9831	0.9861	**0.9868**	0.9865	0.9845	**0.9868**	0.9863	0.9860	0.9842	0.9848	0.9854	**0.9868**	0.9831	**0.9868**	0.9846	0.9815
Test	0.9807	0.9823	**0.9828**	0.9827	0.9806	**0.9828**	0.9826	0.9825	0.9819	0.9821	0.9823	**0.9828**	0.9827	**0.9828**	0.9820	0.9796

D_t_, duplication time in the features enhancement module; N_r_, the number of residual units in the deep feature learning module; N_c_, the number of cells of fully connected neural network layer in deep feature learning module; P_d_, dropout probability.

The duplication time *D_t_* in the feature enhancement module is changed to 1, 5, 10, and 20. As the duplication time increases, the feature information capacity of NCResNet increases. *D_t_* of 10 shows better accuracy on both human training and test datasets. The number of residual units *N_r_* at the deep feature learning module is changed to 1, 3, 5, and 10. The *N_r_* of three shows better performance both on training dataset and test datasets. The number of cells *N_c_* in the fully connected neural network layer is changed to 64, 128, 256, and 512. When *N_c_* is 512, NCResNet achieves the highest accuracy both on the training dataset and test dataset. The dropout probability *P_d_* is changed to 0, 0.3, 0.5, and 0.7. Similar to *N_r_* and *N_c_*, NCResNet achieves higher accuracy when *P_d_* is 0.3 both on the training and test datasets. As a result, we assign *D_t_* = 10, *N_r_* = 3, *N_c_* = 512, and *P_d_* = 0.3 as baseline hyperparameters of our model to balance performance and generalization.

### Performance Comparison Between Tools

To assess the performance of NCResNet, NCResNet is compared with the other five models including CPC2, CPAT, IRSOM, LncFinder, and CPPred. NCResNet is trained on the human training dataset and tested on the eight human, mouse, *S. cerevisiae*, zebrafish, fruit fly, cow, rat and *C. elegans* cross-species test datasets. Because CPAT, IRSOM, and LncFinder provide a retraining interface, they are retrained on the human training dataset same as NCResNet, and tested on the other eight cross-species datasets. Our training dataset is derived from CPPred, so CPPred is not retrained, and we use the default human-CPPred model as the comparison model. Additionally, we use an existing human model of CPC2 to test other cross-species datasets as well because of the lack of a retraining interface. In addition, each species test dataset is split into long-ORF RNA sequence test datasets and short-ORF RNA sequence test datasets based on whether the length of the longest ORF is greater than 303 nt.

As shown in **Table 4**, on the human test dataset with long-ORF RNA sequences, NCResNet outperforms the other models in terms of sensitivity, F1-score, accuracy, AUC, and MCC with 0.9842, 0.9670, 0.9664, 0.9947, and 0.9334, respectively. Although CPPred calls a higher specificity with 0.9540, it shows poor performance in other metrics.

**Table 4:** Performance of NCResNet on the human long-open-reading frame (ORF) RNA sequence test dataset.

	Specificity	Sensitivity	F1-score	Accuracy	AUC	MCC
NCResNet	0.9485	**0.9842**	**0.9670**	**0.9664**	**0.9947**	**0.9334**
CPC2	0.9119	0.9512	0.9152	0.9315	0.9806	0.8637
CPAT	0.9328	0.9575	0.9458	0.9452	0.9835	0.8906
IRSOM	0.9183	0.8983	0.9073	0.9083	0.9083	0.9083
LncFinder	0.9196	0.9595	0.9408	0.9396	0.9803	0.8799
CPPred	**0.9540**	0.9706	0.9626	0.9623	0.9915	0.9247

The human test dataset with short-open-reading frame (ORF) RNA sequences is a challenging dataset that includes 641 ncRNAs and 641 pcRNAs. The test results are listed in **Table 5**. NCResNet calls better performance in terms of specificity, F1-score, accuracy, AUC, and MCC with 0.9329, 0.8357, 0.8494, 0.9323, and 0.7089, respectively, and shows 1.88, 4.5, and 6.04% improvement in terms of specificity, accuracy, and MCC, respectively.

**Table 5:** Performance of NCResNet on the human short-open-reading frame (ORF) RNA sequence test dataset.

	Specificity	Sensitivity	F1-score	Accuracy	AUC	MCC
NCResNet	**0.9329**	0.7659	**0.8357**	**0.8494**	**0.9323**	**0.7089**
CPC2	0.1263	0.9500	0.5209	0.5382	0.7994	0.1348
CPAT	0.3790	0.9703	0.7489	0.6747	0.8496	0.4333
IRSOM	0.9141	0.1653	0.2643	0.5397	0.7256	0.1200
LncFinder	0.1684	0.9734	0.6941	0.5709	0.8382	0.2392
CPPred	0.6271	**0.9797**	0.8328	0.8034	0.9260	0.6485

We also compare NCResNet with other methods by accuracy on cross-species test datasets including mouse, *S. cerevisiae* and zebrafish, fruit fly, cow, rat, and *C. elegans*. NCResNet achieves overwhelming performance on these datasets. As shown in **Table 6**, NCResNet obtains the higher accuracy in mouse, *S. cerevisiae*, zebrafish, cow, rat, and *C. elegans* species with 0.9946, 0.9936, 0.982, 0.985, 0.9815, and 0.9074, respectively, and slightly worse accuracy than CPAT in fruit fly species. on the cross-species test datasets with short-ORF RNA sequences shown in **Table 7**, NCResNet achieves better relative improvement in terms of accuracy compared with cross-species test datasets with long-ORF RNA sequences. Moreover, on the mouse, *S. cerevisiae*, and zebrafish species datasets, the improved accuracy exceeds 10%. 

**Table 6:** Accuracy of six models on cross-species long-open-reading frame (ORF) RNA sequence datasets.

	Mouse	*S. cerevisia*e	Zebrafish	Fruit fly	Cow	Rat	*C. elegans*
NCResNet	**0.9708**	0.9515	0.9463	**0.9702**	**0.926**	**0.9223**	**0.8324**
CPC2	0.9315	0.9563	0.9315	0.9592	0.8503	0.8146	0.6065
CPAT	0.9468	**0.96**	0.9143	0.9297	0.8239	0.6887	0.5867
IRSOM	0.9406	0.9092	0.9203	0.9464	0.8028	0.767	0.6039
LncFinder	0.961	0.9104	0.9259	0.9554	0.875	0.8328	0.6279
CPPred	0.9663	0.9382	**0.9474**	0.9508	0.8926	0.8729	0.7139

**Table 7:** Accuracy of six models on cross-species short-open-reading frame (ORF) RNA sequence datasets.

	Mouse	*S. cerevisiae*	Zebrafish	Fruit-fly	Cow	Rat	*C. elegans*
NCResNet	**0.8321**	**0.8244**	**0.7674**	**0.7335**	**0.8581**	**0.7287**	**0.8888**
CPC2	0.5372	0.5387	0.4599	0.559	0.5544	0.5523	0.5663
CPAT	0.7027	0.5968	0.6201	0.727	0.7862	0.7231	0.7608
IRSOM	0.5017	0.5012	0.4276	0.5354	0.5631	0.5361	0.4959
LncFinder	0.653	0.5326	0.5645	0.702	0.6055	0.5949	0.5743
CPPred	0.7169	0.6779	0.6627	0.5748	0.7043	0.6652	0.8054

**Figure 4:**
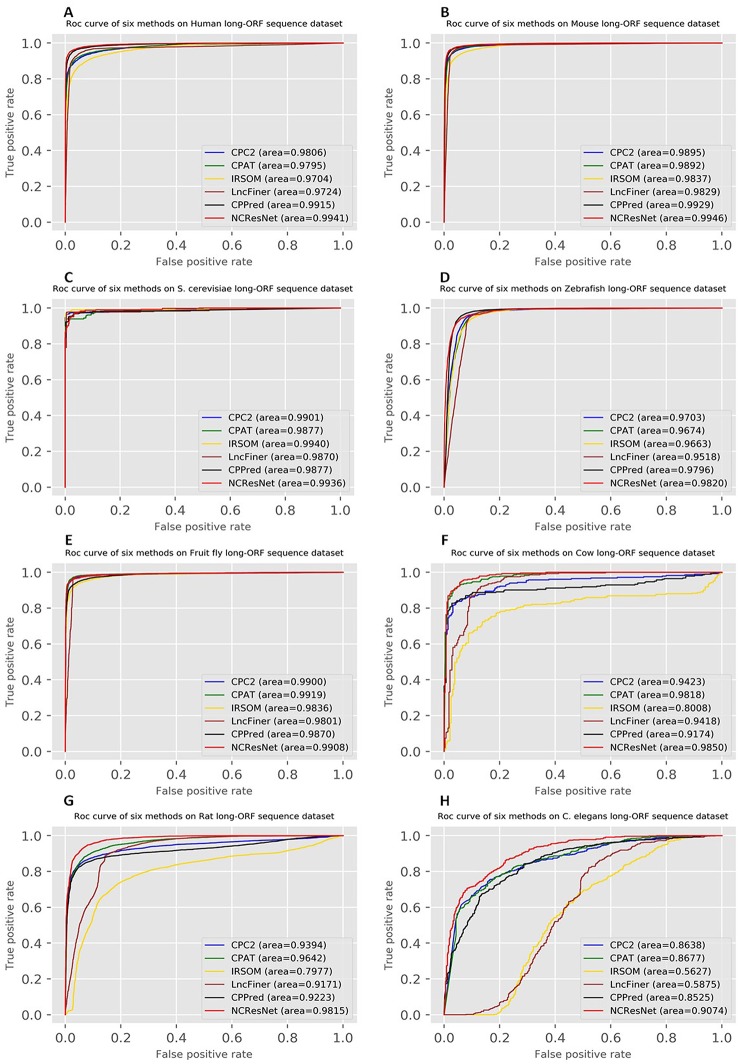
Receiver operating characteristic curves (ROCs) of six methods on cross-species test datasets of human **(A)**, mouse **(B)**, *Saccharomyces cerevisiae* **(C)**, zebrafish **(D)**, fruit fly **(E)**, cow **(F)**, rat **(G)**, and *Caenorhabditis elegans* **(H)** with long-open reading frame (ORF) RNA sequences.

**Figure 5:**
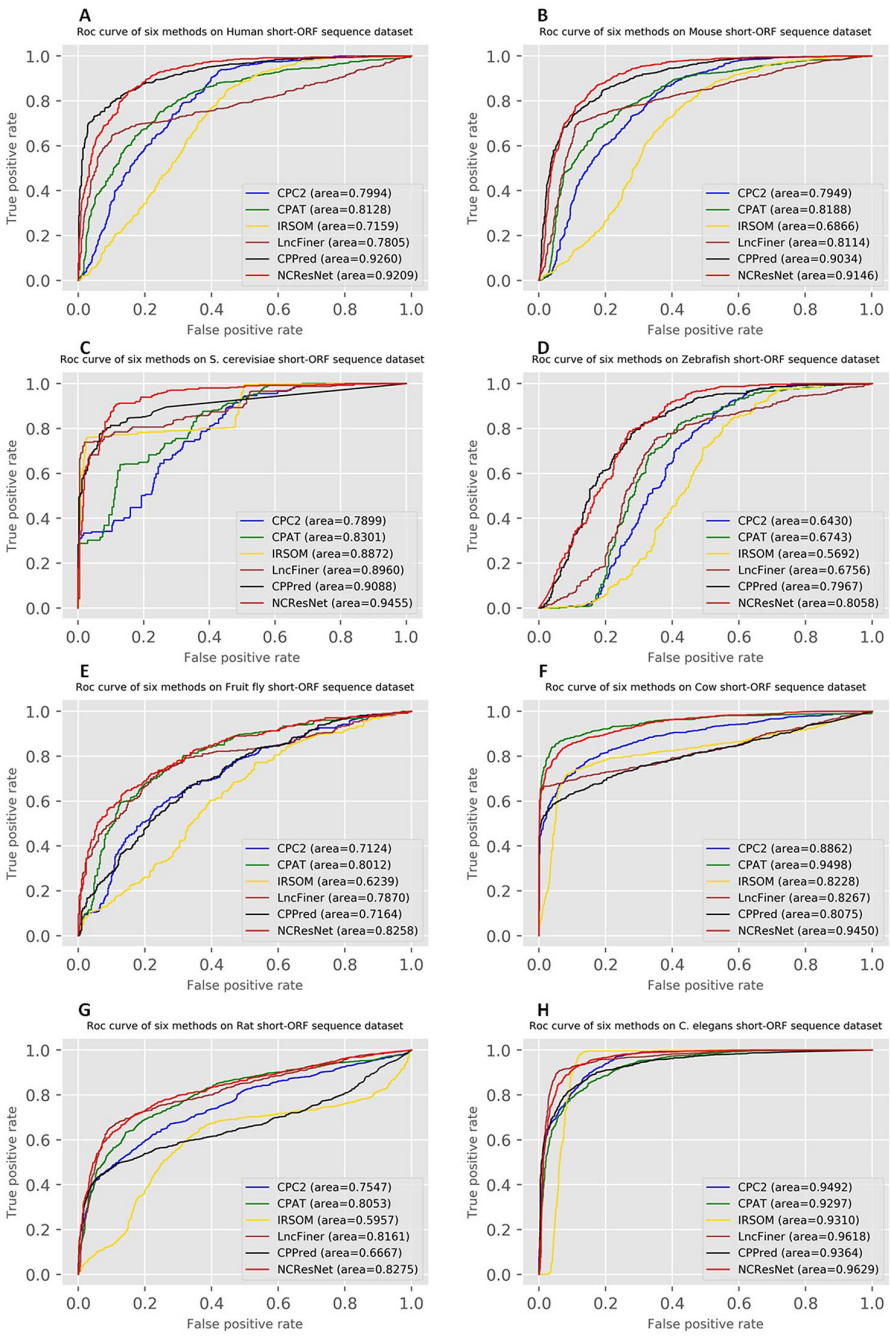
Receiver operating characteristic curves (ROCs) of six methods on cross-species test datasets of human **(A)**, mouse **(B)**, *Saccharomyces cerevisiae* **(C)**, zebrafish **(D)**, fruit fly **(E)**, cow **(F)**, rat **(G)**, and *Caenorhabditis elegans* **(H)** with short-open reading frame (ORF) RNA sequences.

Moreover, the ROCs of six methods in human, mouse, *S. cerevisiae* and zebrafish, fruit fly, cow, rat, and *C. elegans* are drawn. **Figures 4** and **5** show ROCs on cross-species test datasets with long-ORF and short-ORF RNA sequences, respectively. Both on long-ORF RNA sequence test datasets and short-ORF RNA sequence test datasets, NCResNet obtains a higher AUC score on most cross-species datasets.

**Figure 6:**
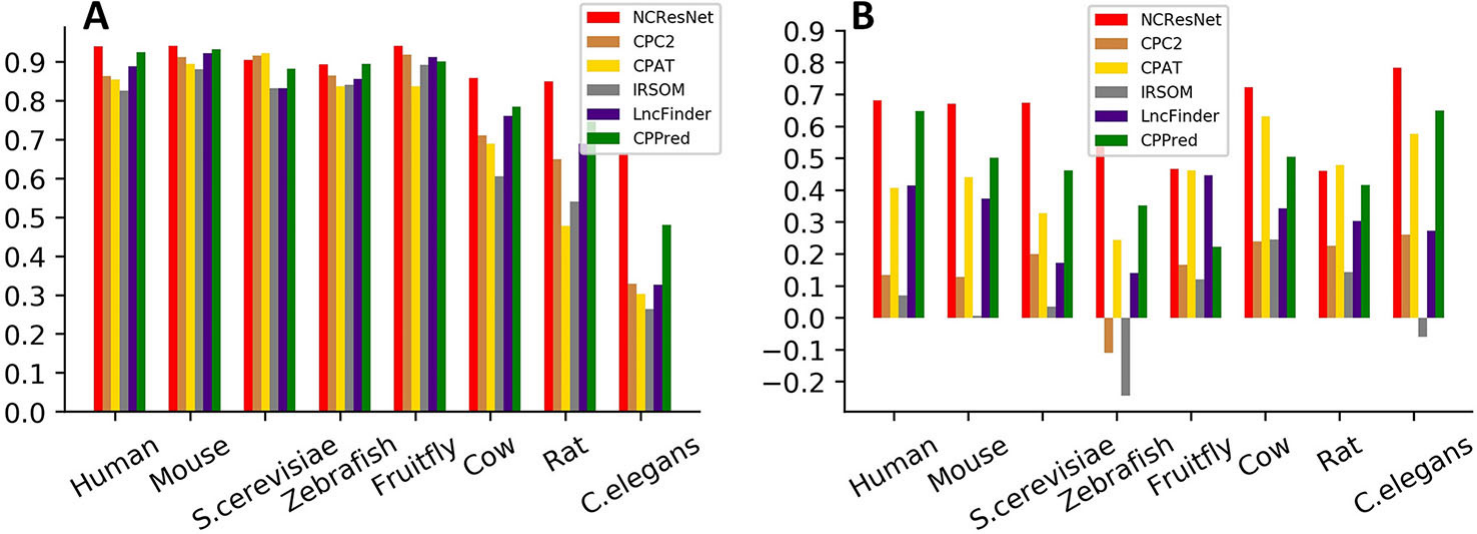
Matthews correlation coefficients (MCCs) of six methods on cross-species test datasets with long-open-reading frame (ORF) test datasets **(A)** and short-ORF **(B)** RNA sequences.

We also employ MCC to measure the performance of six methods. MCC has a range of −1 to 1, where −1 indicates a completely wrong binary classifier, while 1 indicates a completely correct binary classifier. **Figure 6** shows the MCC of each method on cross-species test datasets with long-ORF and short-ORF RNA sequences. On both kinds of datasets, NCResNet obtains a higher MCC value for most species, especially on cross-species datasets with short-ORF RNA sequences.

### Performance Comparison of Models on Verification Datasets

To further evaluate our method, we test the capacity of NCResNet according to the number of correctly predicted ncRNAs and compare it with other methods (CPC2, CPAT, IRSOM, LncFinder, and CPPred). The evaluated data derived from RefLnc, which analyzes a compendium of 14,166 RNA-Seq libraries from 30 physiological tissues, 18 tumors, and 2 cell lines from two independent datasets, obtain 27,520 robust novel lncRNAs. Among the 27,520 lncRNAs are 20,364 long-ORF ncRNA sequences and 7,142 short-ORF ncRNA sequences. From **Table 8**, we can see that NCResNet correctly predicts all long-ORF ncRNA sequences and all short-ORF ncRNA sequences.

**Table 8:** Number of noncoding RNA (ncRNAs) correctly predicted by six models on the verification datasets.

Data type	Number of new data	NCResNet	CPC2	CPAT	IRSOM	LncFinder	CPPred
Long-ORF ncRNAs	20,364	**20,364**	19,787	19,487	19,341	19,703	20,112
Short-ORF pcRNAs	7,142	**7,142**	7,141	7,105	**7,142**	7,828	7,111

### Performance Comparison of Six Models on Integrated Datasets

Afterward, we merge the above eight popular species datasets into an integrated dataset for the sake of eliminating the problems caused by the specificity of species and the differences between the databases. We select two-thirds RNA sequences as an integrated-training dataset randomly and the rest as an integrated-test dataset. In addition, the integrated-test dataset is split into the long-ORF RNA sequence dataset and short-ORF RNA sequence dataset. In view of CPC2 and CPPred do not provide retraining codes, we train NCResNet and retrain CPAT, IRSOM, and LncFinder on the integrated-training dataset and compare them on integrated-test datasets. **Tables 9** and **10** show the performance of the compared methods on the integrated-test dataset with long-ORF and short-ORF RNA sequences, respectively, and indicate that both on the long-ORF and short-ORF RNA sequence integrated-test datasets, NCResNet achieves better performance in terms of F1-score, accuracy, AUC, and MCC. Particularly on integrated-test datasets with short-ORF RNA sequences, NCResNet performs much better than the other compared methods with more than 3.53, 5.15, 6.8, and 7.21% improvement in F1-score, accuracy, AUC, and MCC, respectively.

**Table 9:** Performance of four models on the integrated-test dataset with long-open reading frame (ORF) RNA sequences.

	Specificity	Sensitivity	F1-score	Accuracy	AUC	MCC
NCResNet	0.9542	0.9059	**0.9283**	**0.9301**	**0.9760**	**0.8611**
CPAT	0.9558	0.7095	0.8091	0.8326	0.9381	0.6864
IRSOM	0.5199	**0.9457**	0.7421	0.7087	0.9020	0.4986
LncFinder	**0.9697**	0.7478	0.8412	0.8588	0.9380	0.7360

**Table 10:** Performance of four models on the integrated-test dataset with short-open reading frame (ORF) RNA sequences.

	Specificity	Sensitivity	F1-score	Accuracy	AUC	MCC
NCResNet	0.7663	0.9673	**0.8790**	**0.8668**	**0.9705**	**0.7490**
CPAT	0.1595	0.9948	0.7017	0.5771	0.8593	0.2808
IRSOM	**0.9462**	0.2813	0.4320	0.4688	0.8160	0.2483
LncFinder	0.6339	**0.9968**	0.8437	0.8153	0.9025	0.6769

### Running Time Comparison of Six Models

We also compare the efficiency of six methods according to the average consuming time of per sequence on a 10,000-sequence dataset. First, we select 10,000 long-ORF RNA sequences (including 5,000 ncRNAs and 5,000 pcRNAs) and 10,000 short-ORF RNA sequences (including 5,000 ncRNAs and 5,000 pcRNAs) from test datasets randomly. Then, we record the average cost time of per sequence of six methods on a 10,000 long-ORF RNA sequence dataset and a 10,000 short-ORF RNA sequence dataset. On 10,000 long-ORF RNA sequence dataset, NCResNet average running time of per sequence is 0.0112 s (0.0112 s) *versus* CPC2 (0.0013 s), CPAT (0.0017 s), IRSOM (0.0012 s), LncFinder (0.0030 s), and CPPred (0.0401 s). And, on 10,000 short-ORF RNA sequence dataset, NCResNet average running time of per sequence is 0.0049 s *versus* CPC2 (0.0005 s), CPAT (0.0004 s), IRSOM (0.0011 s), LncFinder (0.0019 s), and CPPred (0.0091 s). **Table 11** lists the average running time of per sequence comparison and **Table 12** shows the corresponding accuracy. Although the running time of per sequence of NCResNet is larger than CPC2, CPAT, IRSOM, LncFinder, NCResNet achieve higher accuracy and the corresponding total running time is in the second level which is within an acceptable range. Additionally, the hardware device of the running environment is CPU (i7-7700, 3.6 GHz), memory (8 G, 2,300 Hz).

**Table 11:** Comparison of six methods average running time of per sequence on a 10,000 long-open reading frame (ORF) RNA sequence dataset and a 10,000 short-ORF RNA sequence dataset.

	NCResNet	CPC2	CPAT	IRSOM	LncFinder	CPPred
Long-ORF sequence dataset	0.0112	0.0013	0.0017	0.0012	0.0030	0.0401
Short-ORF sequence dataset	0.0049	0.0005	0.0004	0.0011	0.0019	0.0091

**Table 12:** Corresponding accuracy of six methods average running time of per sequence on a 10,000 long-open reading frame (ORF) RNA sequence dataset and a 10,000 short-ORF RNA sequence dataset.

	NCResNet	CPC2	CPAT	IRSOM	LncFinder	CPPred
Long-ORF sequence dataset	0.9662	0.9506	0.9402	0.9353	0.9559	0.9649
Short-ORF sequence dataset	0.8171	0.5455	0.7169	0.5150	0.5254	0.7207

## Discussion

Deep learning technology has yielded inspiring results for many issues related to bioinformatics owing to the increase in training data and relatively complex neural network structure (Min et al., 2017). The issue of distinguishing ncRNAs from pcRNAs is a vital and indispensable step to explore the functions of novel RNAs. With the rapid development of next-generation sequencing technology, numerous novel RNAs have been generated. However, the differentiation of ncRNAs from pcRNAs by biological experiments is expensive and time-consuming. Previous computational methods have achieved excellent performance on long-ORF RNA sequences, but most of them do not adapt to short-ORF RNA sequences. In this paper, NCResNet is proposed to fill this gap by multiple hybrid features and deep-learning-based structure.

NCResNet introduces 57 hybrid features of four categories, including RNA sequence, RNA structure, protein and RNA physicochemical property. An RNA sequence can be characterized comprehensively, based on the 57 hybrid features combination of four categories. For example, if the RNA-sequence-based features, e.g., features related to ORFs, poorly support the recognition of the ncRNA, other multiple hybrid category features will fill the gap. Moreover, we do not employ feature engineering to find and select powerful and contributed features because models based on the deep neural network are not sensitive to a few less contributed features due to the automatic parameter learning. Therefore, the performance of NCResNet is affected very slightly by some features that are not the best candidates.

NCResNet consists of four modules: an input module, a features enhancement module, a deep feature learning module and a prediction module. The 57 hybrid features calculated from the input model are mapped into a higher dimensional space by the repeat and flatten layers in the feature enhancement module, and then the generated enhancement feature information will be compressed to a relatively lower dimension vector by the compression unit in the deep feature learning module. These processes can contribute and strengthen candidate feature information and reduce the adverse effects of a few slightly contributing features. Furthermore, stacked residual units in the deep feature learning module are introduced to learn and detect high-level features. Although the residual unit can effectively overcome gradient vanishing and the gradient explosion problem, it faces another issue: the loss of useful information from original features. To solve this problem, two integration units in the deep feature learning module are utilized to fuse the high-level features from the residual units and the primary features from the compression unit.

Due to its multiple hybrid features and deep-learning-based structure, NCResNet achieves excellent performance on both long-ORF and short-ORF RNA sequence datasets. However, the core structure of NCResNet, a deep neural network structure, is a black box, and it is hard and difficult to interpret the performance and evaluate the importance of every input feature. Therefore, more efforts are needed to explore the interpretability of NCResNet, which will help us discover more novel characteristics of ncRNAs.

## Conclusion

In this work, we propose a deep learning-based method, NCResNet, to identify ncRNA by using 57 hybrid features of four categories, which are derived from sequences, protein, RNA structure, and RNA physicochemical property. NCResNet consists of four main modules: an input module, a feature enhancement module, a deep feature learning module, and a prediction module. Based on the feature enhancement and deep feature learning policies, NCResNet can learn more contributed and useful feature information. As a result, on short-ORF RNA sequence test datasets including species such as mouse, *S. cerevisiae*, zebrafish, fruit fly, and cow, NCResNet achieves more than 10 and 15% improvement over the compared methods in terms of accuracy and MCC, respectively. Meanwhile, on long-ORF RNA sequence test datasets, NCResNet achieves higher accuracy and higher MCC than other methods on most species datasets. Overall, NCResNet successfully detects short-ORF ncRNA sequences and shows robust performance on long-ORF RNA sequence datasets as well, and our method will contribute to the identification of novel ncRNAs from abundant transcriptome data.

## Data Availability Statement

Publicly available datasets were analyzed in this study. Codes and data are available here: https://github.com/abcair/NCResNet.

## Author Contributions

SY, YW, and XH wrote the manuscript. SY and SZ constructed NCResNet model. SY and QM collected and cleaned the collected data. YW, YT, and QM edited the manuscript.

## Funding

This research was funded by the National Natural Science Foundation of China (Nos. 61872418, 61972174, 61902144), the Development Project of Jilin Province of China (Nos. 20170203002GX, 20170520063JH, 20180414012GH, 20190201293JC), Guangdong Key-Project for Applied Fundamental Research (Grant 2018KZDXM076) and Collaborative Innovation Center for Big Data Applications of JLUZH (Grant 2018XJCQ008).

## Conflict of Interest

The authors declare that the research was conducted in the absence of any commercial or financial relationships that could be construed as a potential conflict of interest.
