# Supplementary material for: NCResNet: Noncoding Ribonucleic Acid Prediction Based on a Deep Resident Network of Ribonucleic Acid Sequences
Source: Front Genet. 2020 Feb 28;11:90. doi: 10.3389/fgene.2020.00090 (PMC7059790; doi:10.3389/fgene.2020.00090)
Supplement: Supplementary file 1 [file DataSheet_1.docx]

Supplementary Material

**Supplementary Table 1. Feature name and Definition**

|  | **Number** | **Feature Name** | **Definition** |
| --- | --- | --- | --- |
| Sequence features | 1 | STOP Codon Number | The number of STOP Codon in the transcript |
|  | 2 | STOP Codon Frequency | STOP Codon Frequency in the transcript |
|  | 3 | ORF Coverage | Ratio of ORF length and transcript length |
|  | 4 | Transcript Length | The length of transcript |
|  | 5 | ORF Length | The length of ORF |
|  | 6 | ORF Integrity | Whether the ORF starts with a START Codon and ends with a STOP Codon |
|  | 7 | Ficket TESTCODE Score | $N_{content}$= Frequency of nucleotide N    $N_{position}=\frac{Max(N_{i})}{Min(N_{i})} i\in\{1,2,3\}$ $\# N_{i}$= Number of nucleotide N at frame $i$ $Fickett Score=\sum_{i=1}^{8} p_{n}w_{n}$    #$p_{n}$= probability of $N_{content}$or $N_{position}$determined by a look up table  # $w_{n}$=weight of eight probabilities $p_{n}$ |
|  | 8 | Hexamer | Hexamer Score = $Hexamer Score =\frac{1}{m}\sum_{i=1}^{m} \log\left(\frac{F(H_{i})}{F^{'}(H_{i})}\right)$  #$F(H_{i}) (i=0,1,...,4095)$and $F^{'}(H_{i}) (i=0,1,...,4095)$ represent in-frame hexamer frequency |
|  | 9 | GC1 | GC content in the first position of codons |
|  | 10 | GC2 | GC content in the second position of codons |
|  | 11 | GC3 | GC content in the third position of codons |
|  | 12 | GC1 Frame Score | Variance of GC1 among three reading frames |
|  | 13 | GC2 Frame Score | Variance of GC2 among three reading frames |
|  | 14 | GC3 Frame Score | Variance of GC3 among three reading frames |
|  | 15 | Seq.Dist.Ratio | Ratio of Dist.NC and Dist.PC |
| Protein features | 16 | Molecular Weight | Molecular weight of a predicted peptide calculated by the ProtParam module in BioPython |
|  | 17 | Point Isoelectric | Theoretical isoelectric point of a predicted peptide calculated by the ProtParam module in BioPython |
|  | 18 | Gravy | Grand average of hydropathicity of a predicted peptide calculated by the ProtParam module in BioPython |
|  | 19 | Instability Index | An estimation of the stability of a predicted peptide in a test tube calculated by the ProtParam module in BioPython |
|  | 20 | Ratio of Point isoelectric and Molecular Weight | Ratio of pI and Mw |
|  | 21 | Variance of ratio of Point isoelectric and Molecular Weight | Variance of pI/Mw among three reading frames |
| RNA Physicochemical property features | 22 | Singal.Peak | Peak power spectrum value of transcript |
|  | 23 | SNR | signal-to-noise ratio |
|  | 24 | Singal.Min | Minimum power value in sorted power spectrum |
|  | 25 | Singal.Q1 | lower quartile power value in sorted power spectrum |
|  | 26 | Singal.Q2 | upper quartile power value in sorted power spectrum |
|  | 27 | Singal.Max | Maximum value of sorted power spectrum |
| Composition, Transition and Distribution features | 28 | A | Composition：composition features are the number of amino acids of a particular property divided by the total number of amino acids. |
|  | 29 | T |  |
|  | 30 | G |  |
|  | 31 | C |  |
|  | 32 | AT | Transition：transition features characterize the percent frequency with which amino acids of a particular property are followed by amino acids of a different property. |
|  | 33 | AG |  |
|  | 34 | AC |  |
|  | 35 | TG |  |
|  | 36 | TC |  |
|  | 37 | GC |  |
|  | 38 | A0 | Distribution：distribution features measure the chain length within which the first, 25%, 50%, 75% and 100% of the amino acids of a particular property are located |
|  | 39 | A1 |  |
|  | 40 | A2 |  |
|  | 41 | A3 |  |
|  | 42 | A4 |  |
|  | 43 | T0 |  |
|  | 44 | T1 |  |
|  | 45 | T2 |  |
|  | 45 | T3 |  |
|  | 47 | T4 |  |
|  | 48 | C0 |  |
|  | 49 | C1 |  |
|  | 50 | C2 |  |
|  | 51 | C3 |  |
|  | 52 | C4 |  |
|  | 53 | G0 |  |
|  | 54 | G1 |  |
|  | 55 | G2 |  |
|  | 56 | G3 |  |
|  | 57 | G4 |  |

**Supplementary Table 2. Features density distribution in Human training datasets**

|  | **Number** | Feature name | Density distribution in Human training datasets |
| --- | --- | --- | --- |
| Sequence features | 1 | STOP Codon Number | 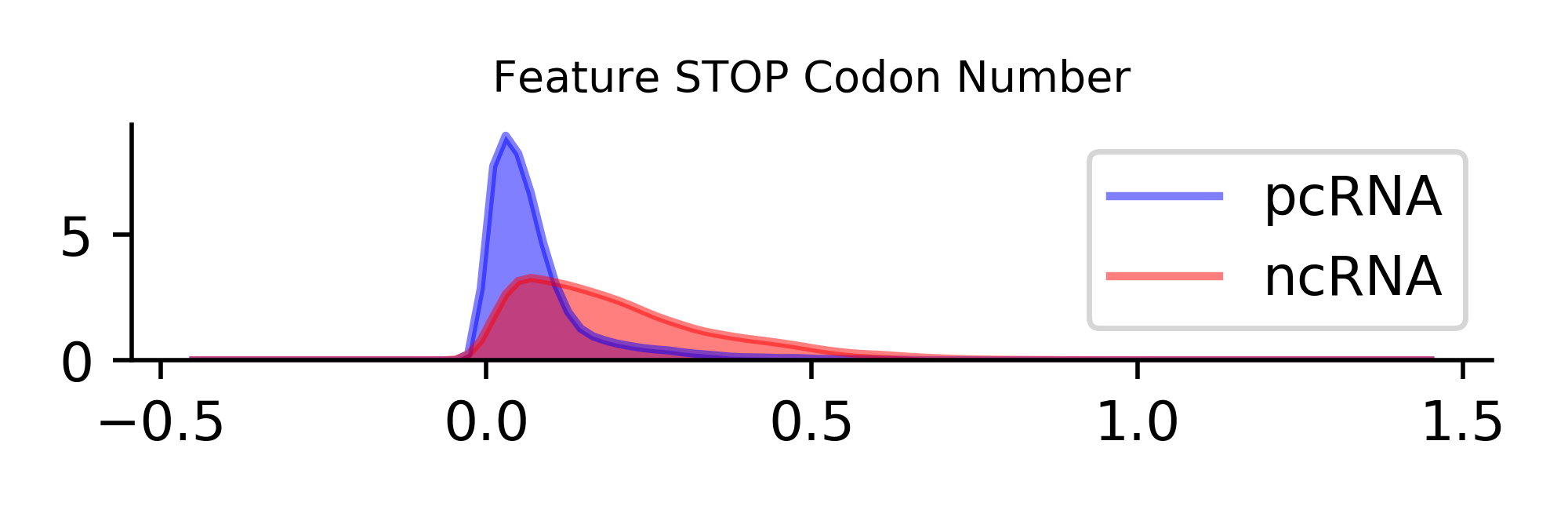 |
|  | 2 | STOP Codon Frequency | 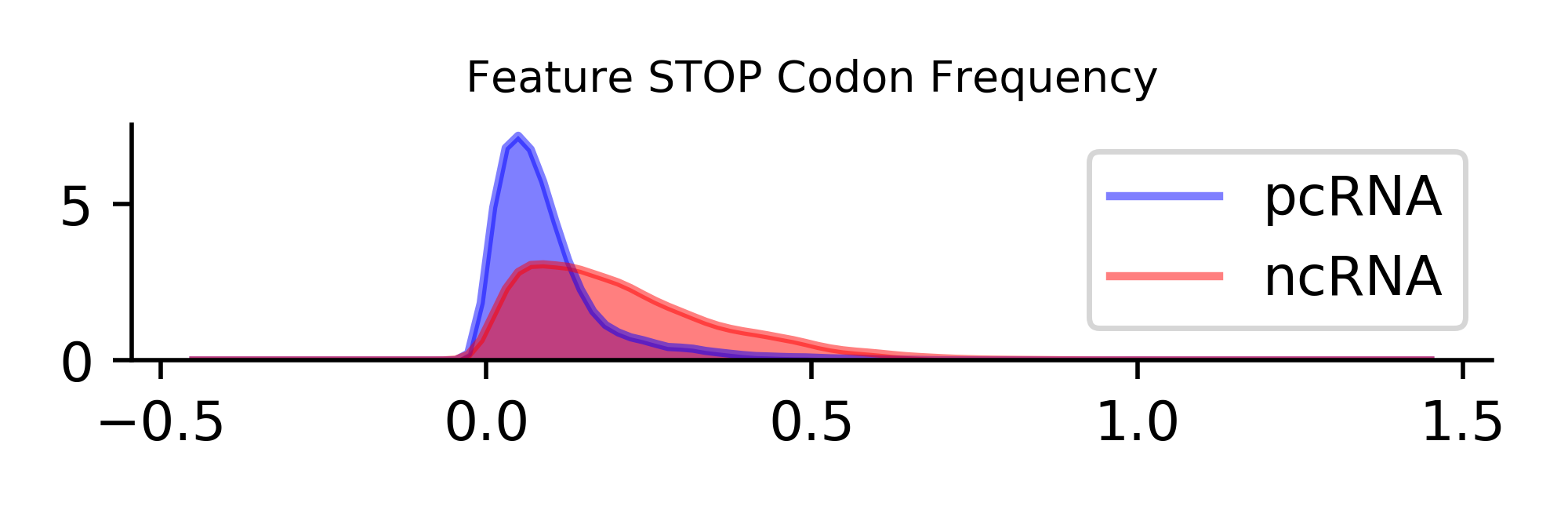 |
|  | 3 | ORF Coverage | 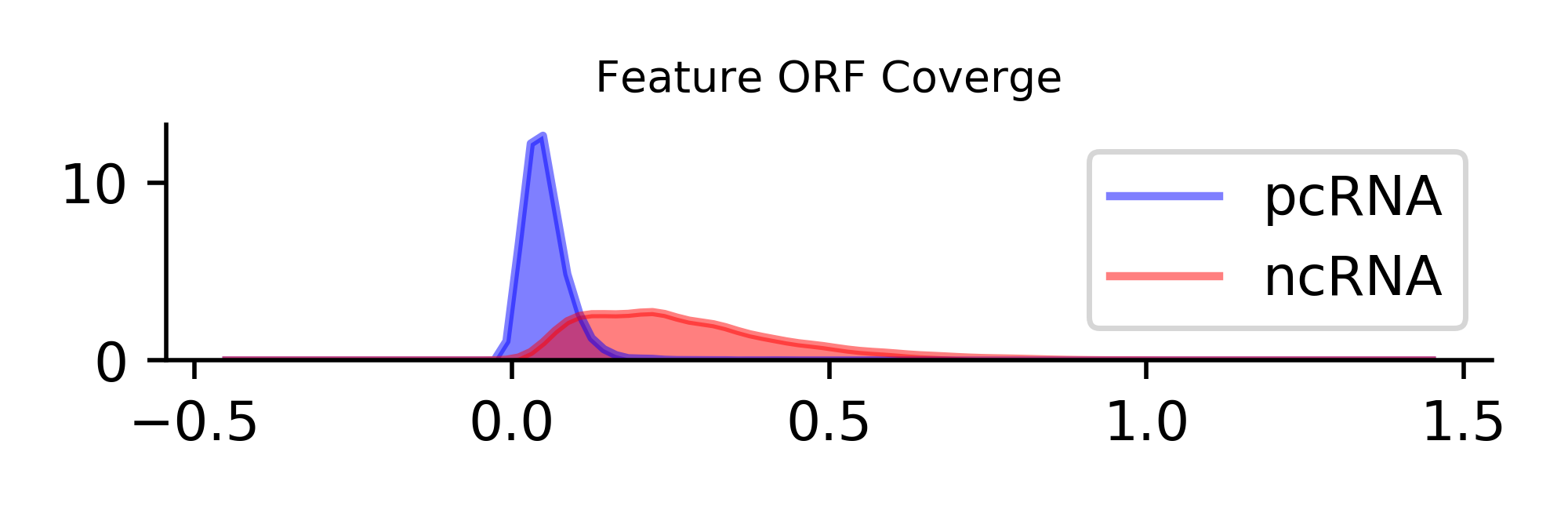 |
|  | 4 | Transcript Length | 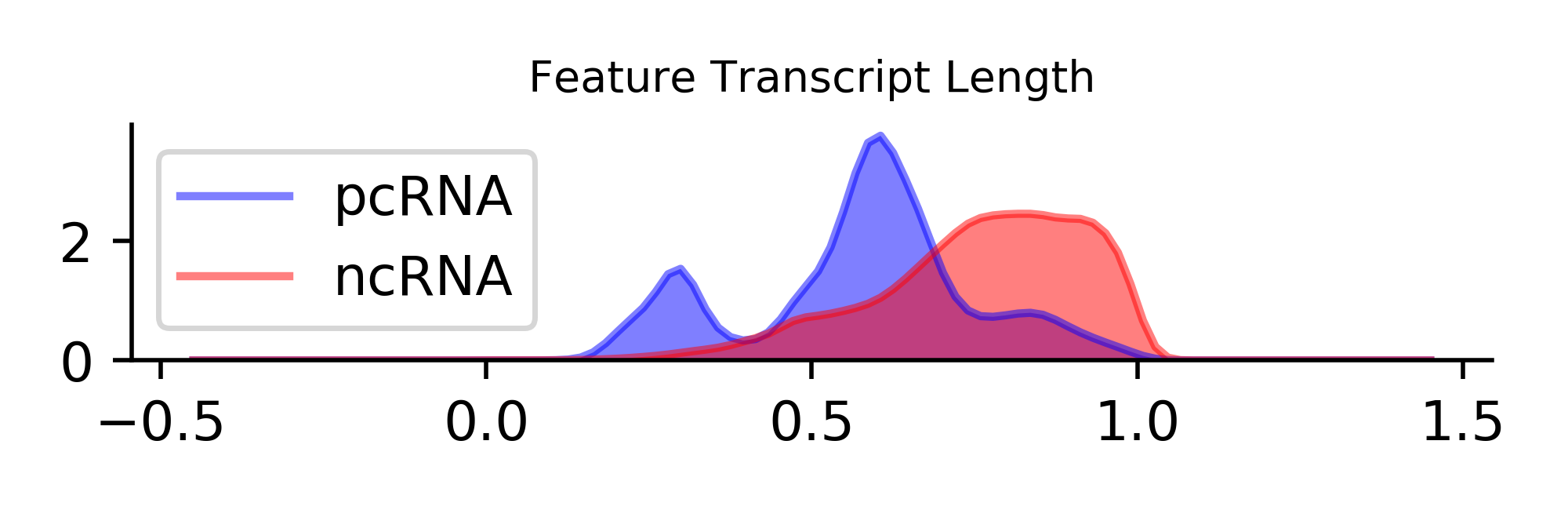 |
|  | 5 | ORF Length | 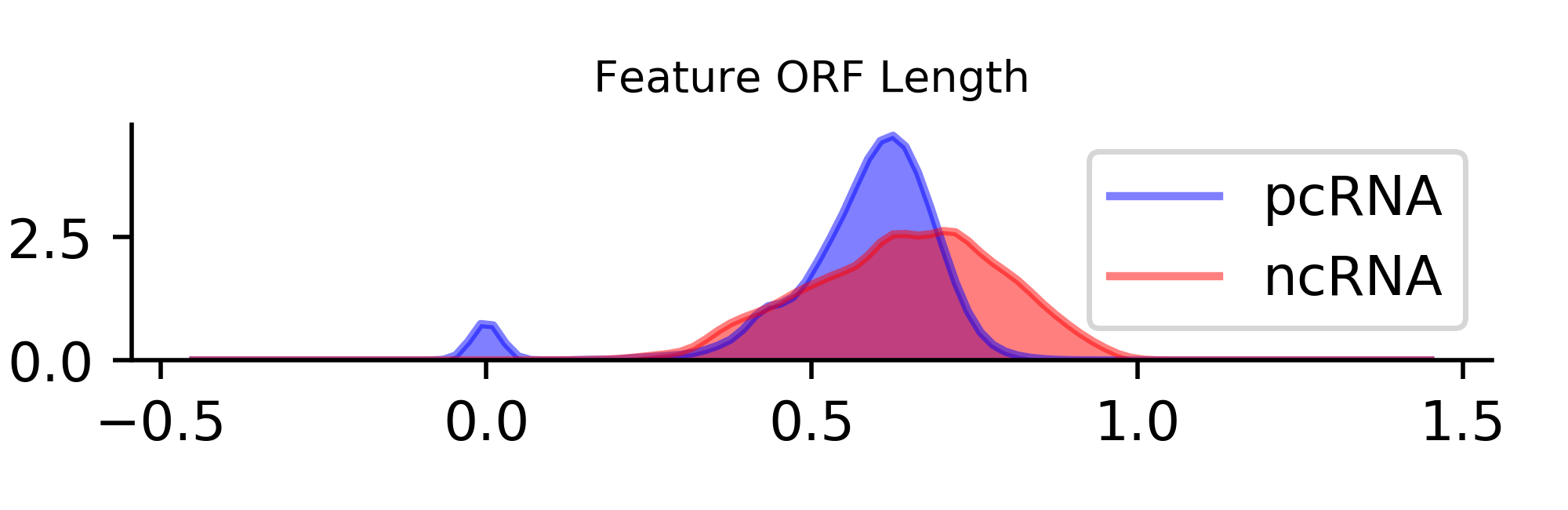 |
|  | 6 | ORF Integrity | 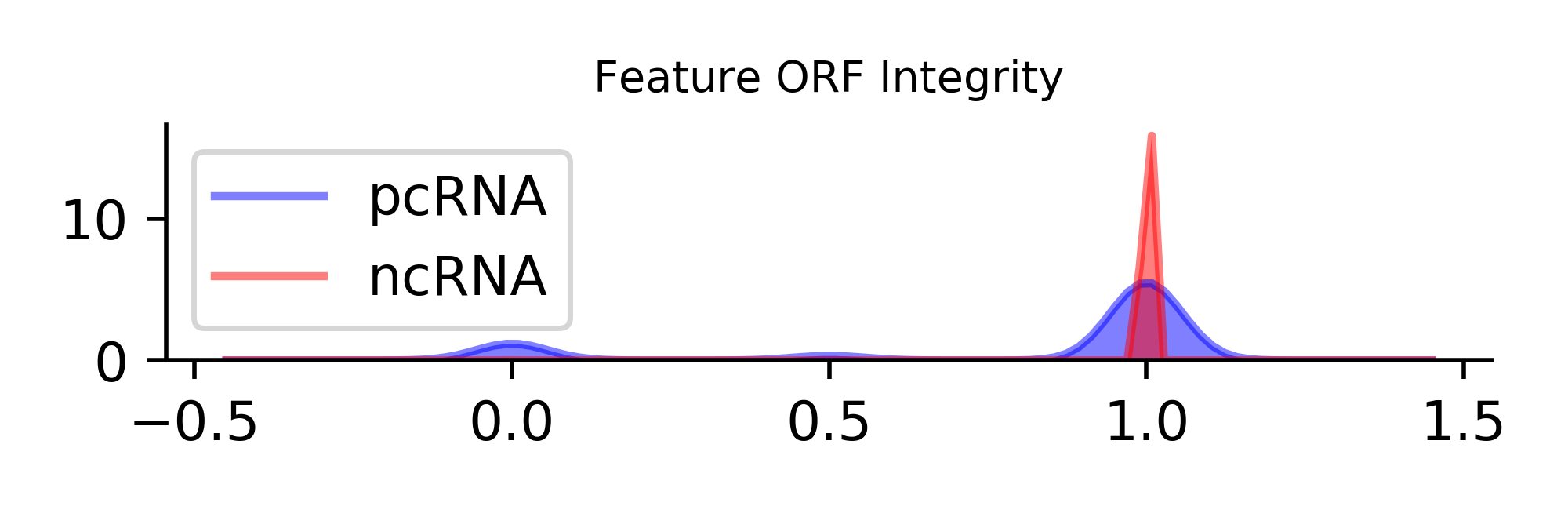 |
|  | 7 | Ficket TESTCODE Score | 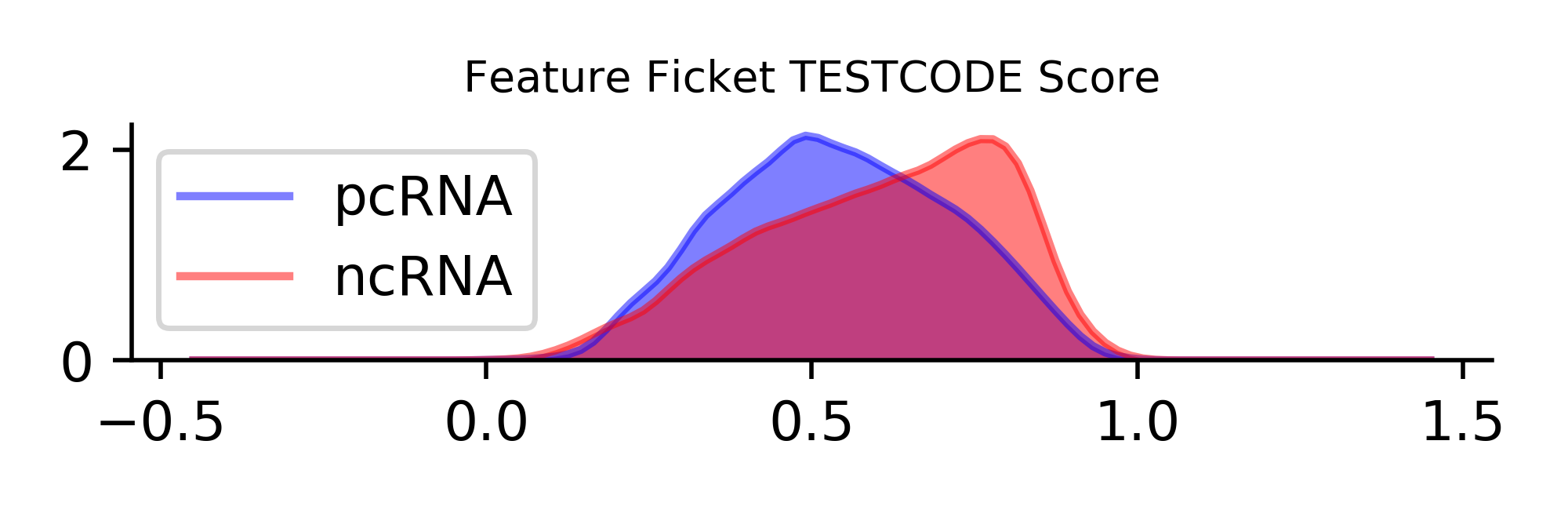 |
|  | 8 | Hexamer | 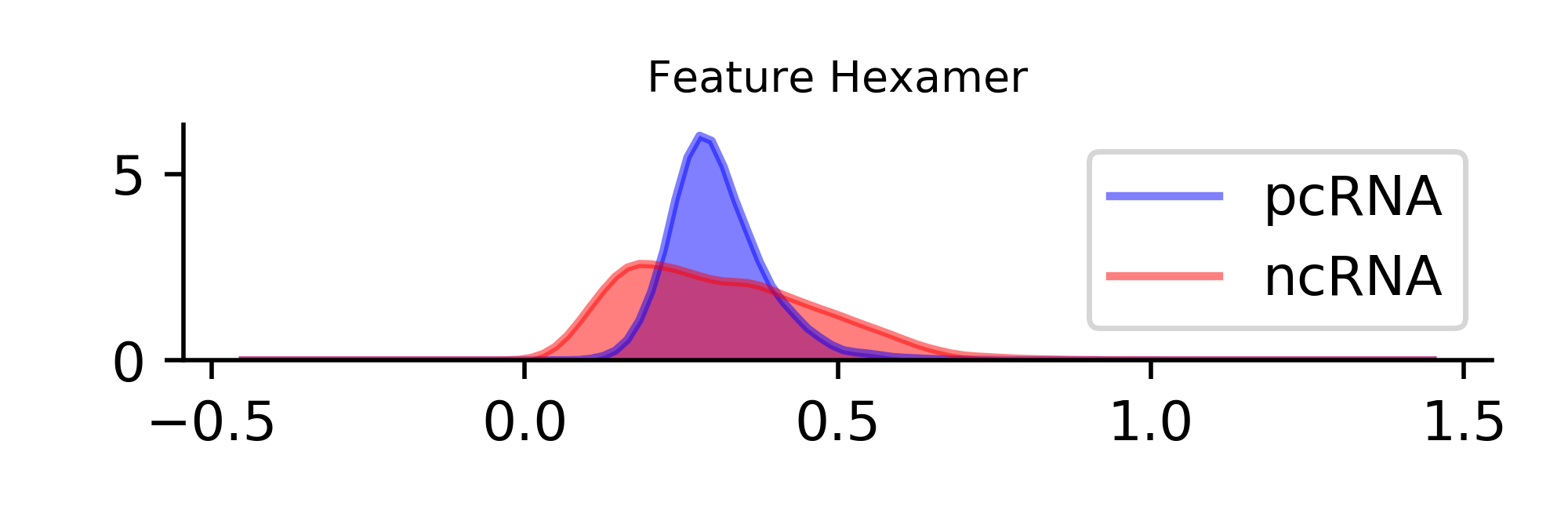 |
|  | 9 | GC1 | 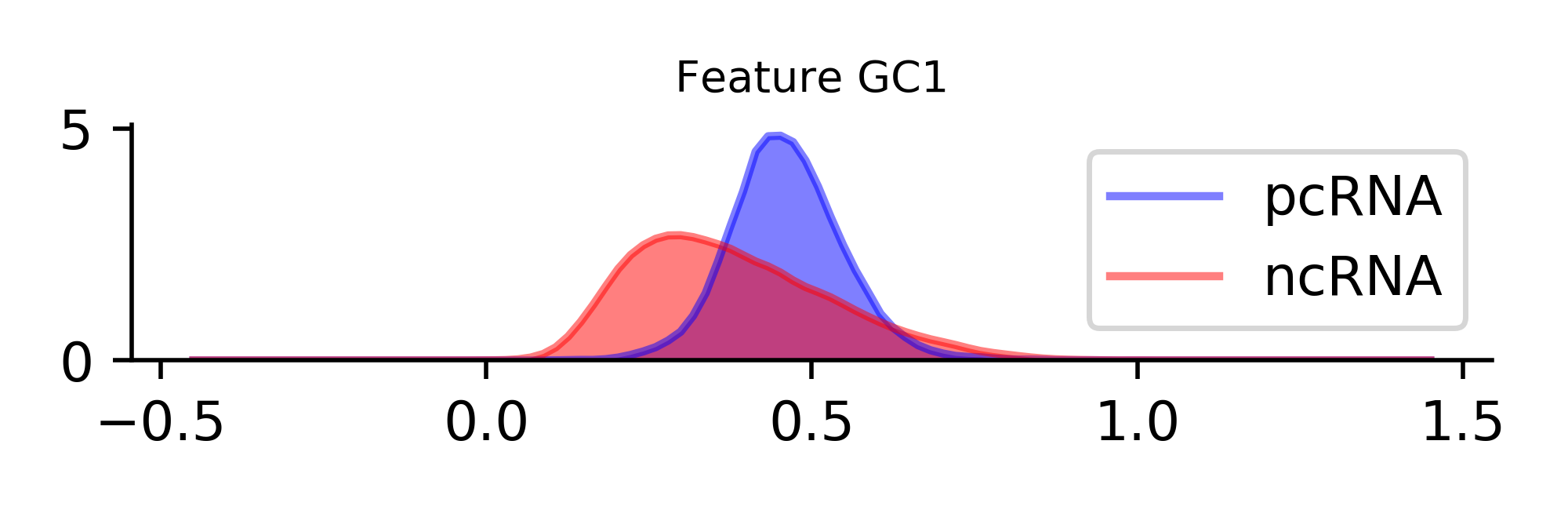 |
|  | 10 | GC2 | 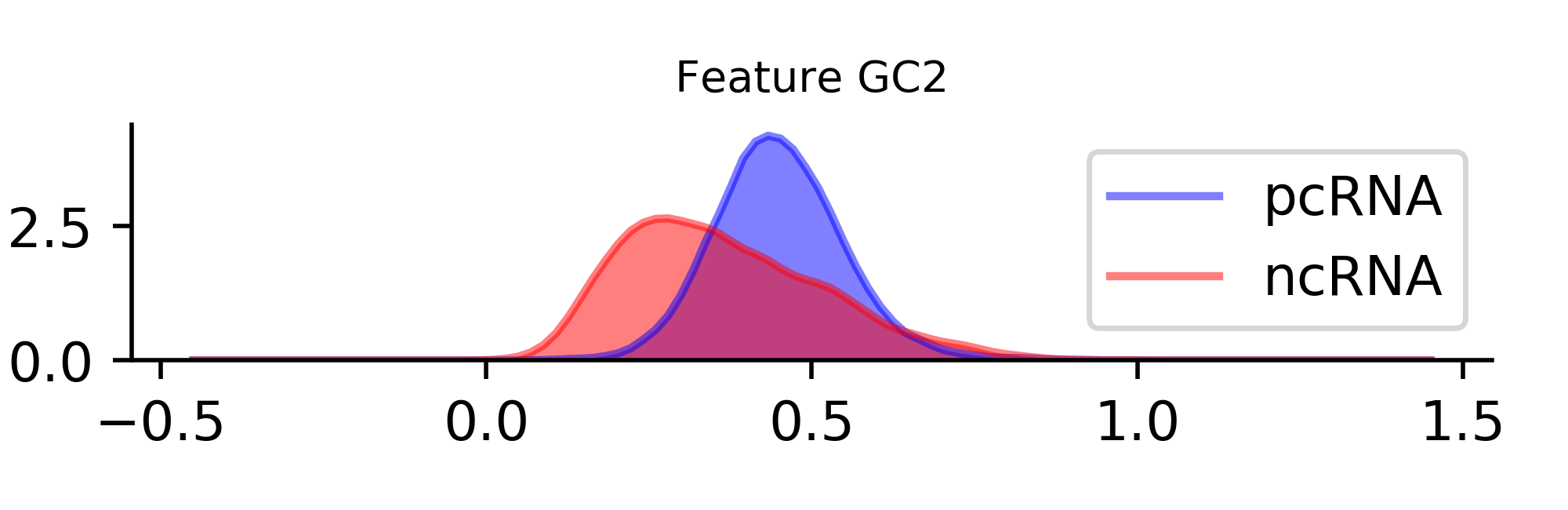 |
|  | 11 | GC3 | 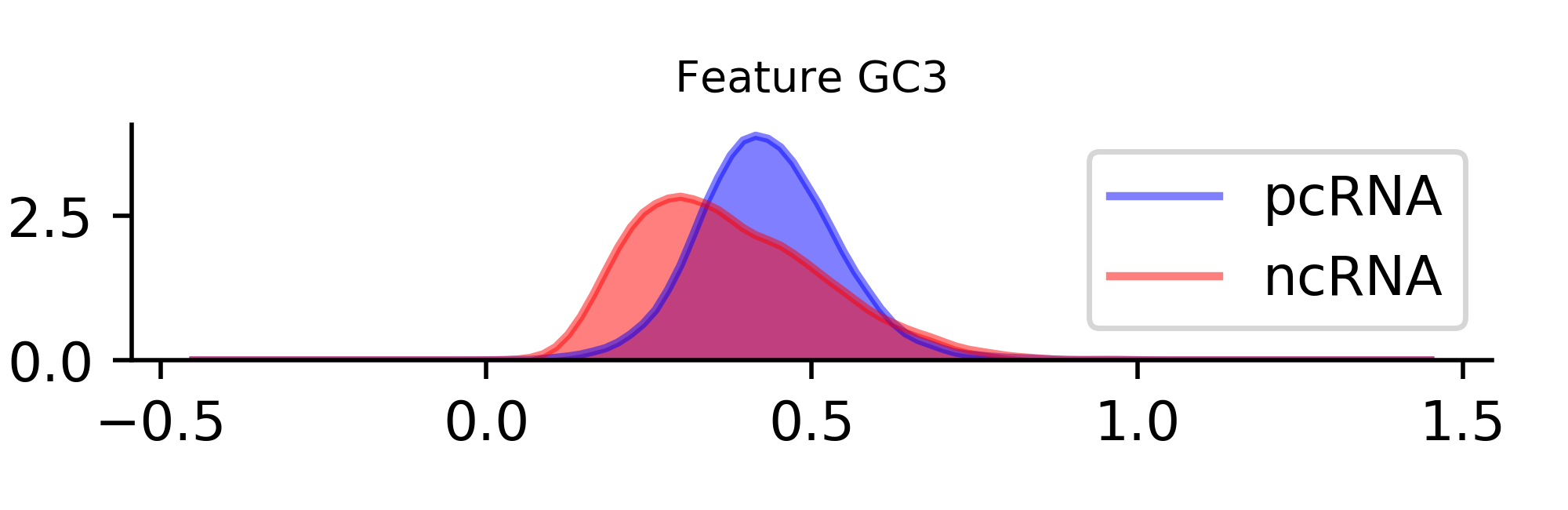 |
|  | 12 | GC1 Frame Score | 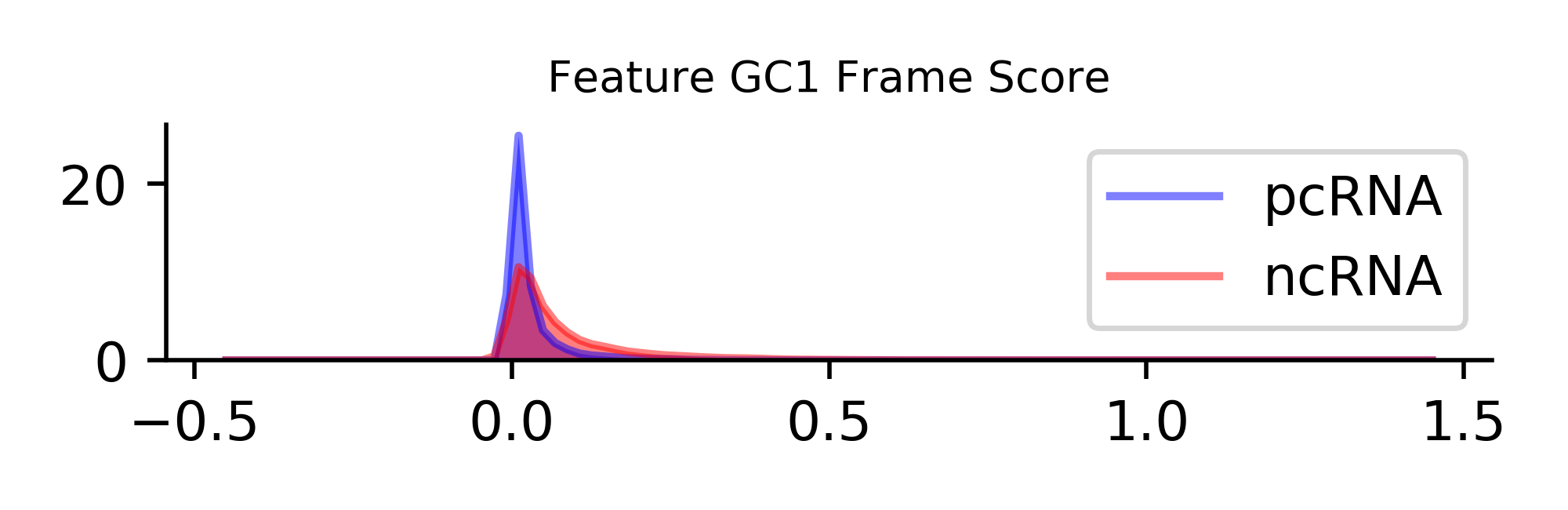 |
|  | 13 | GC2 Frame Score | 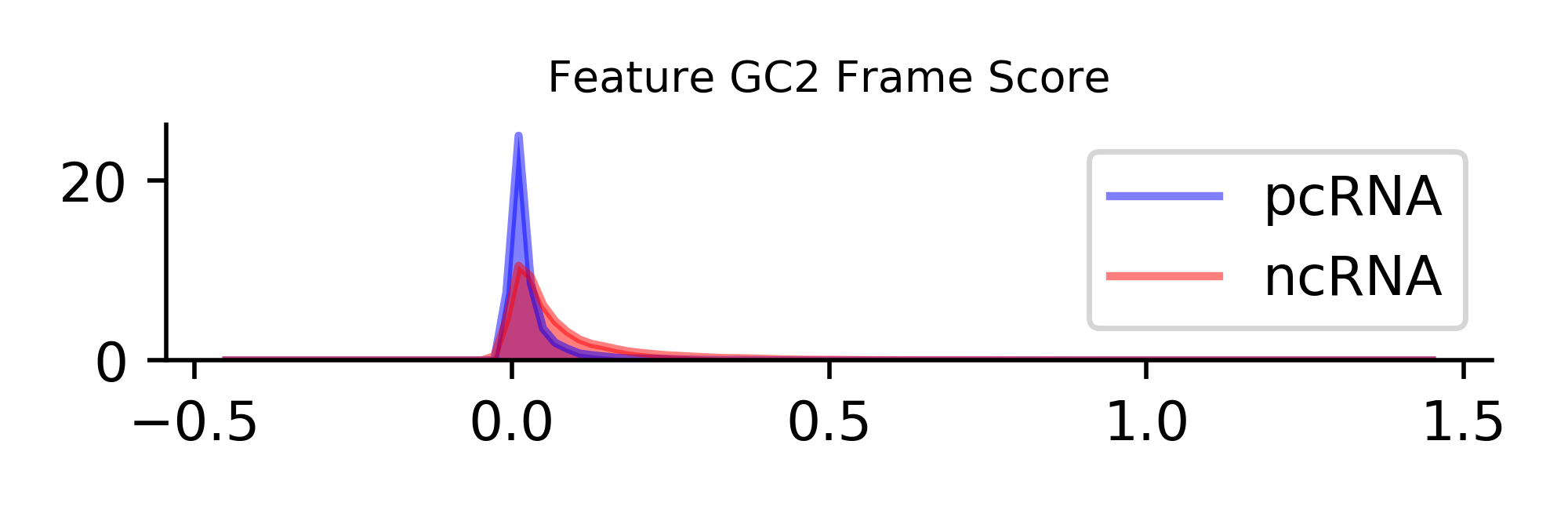 |
|  | 14 | GC3 Frame Score | 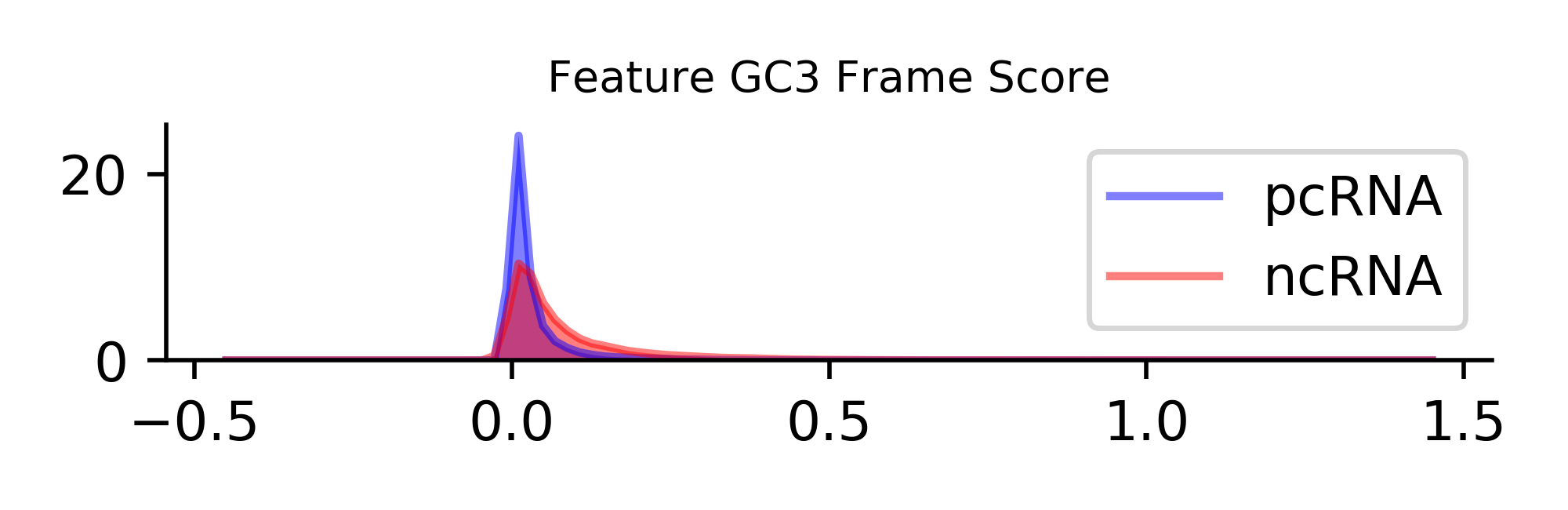 |
|  | 15 | Seq.Dist.Ratio | 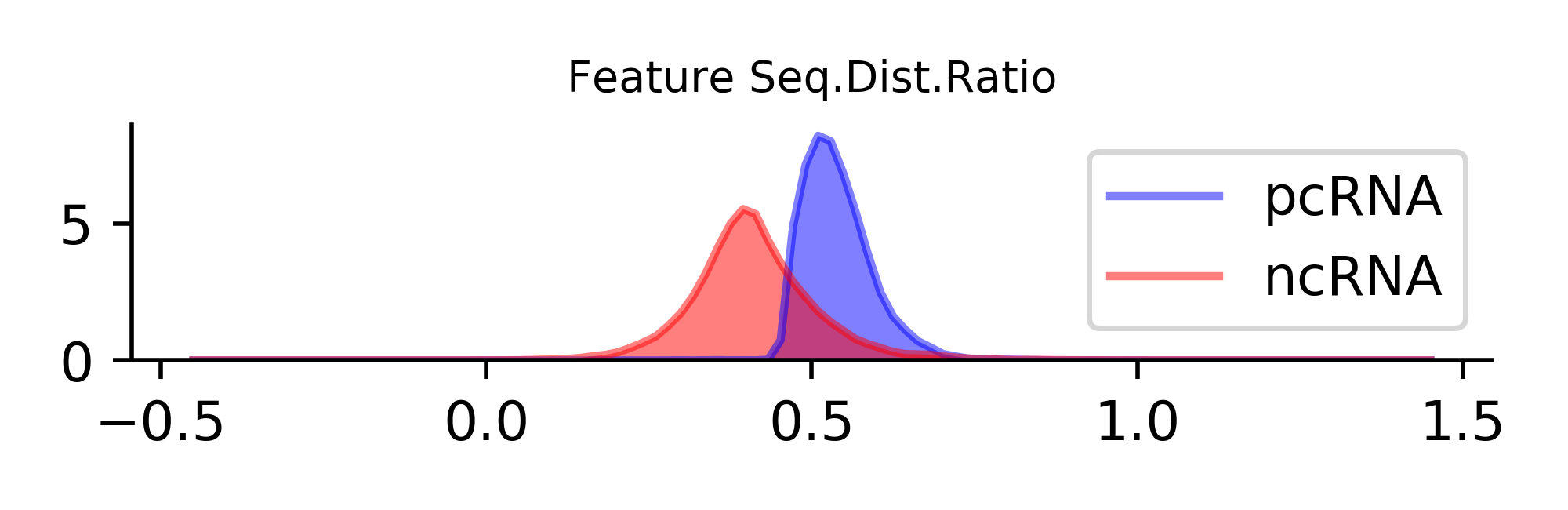 |
| Protein features | 16 | Molecular Weight | 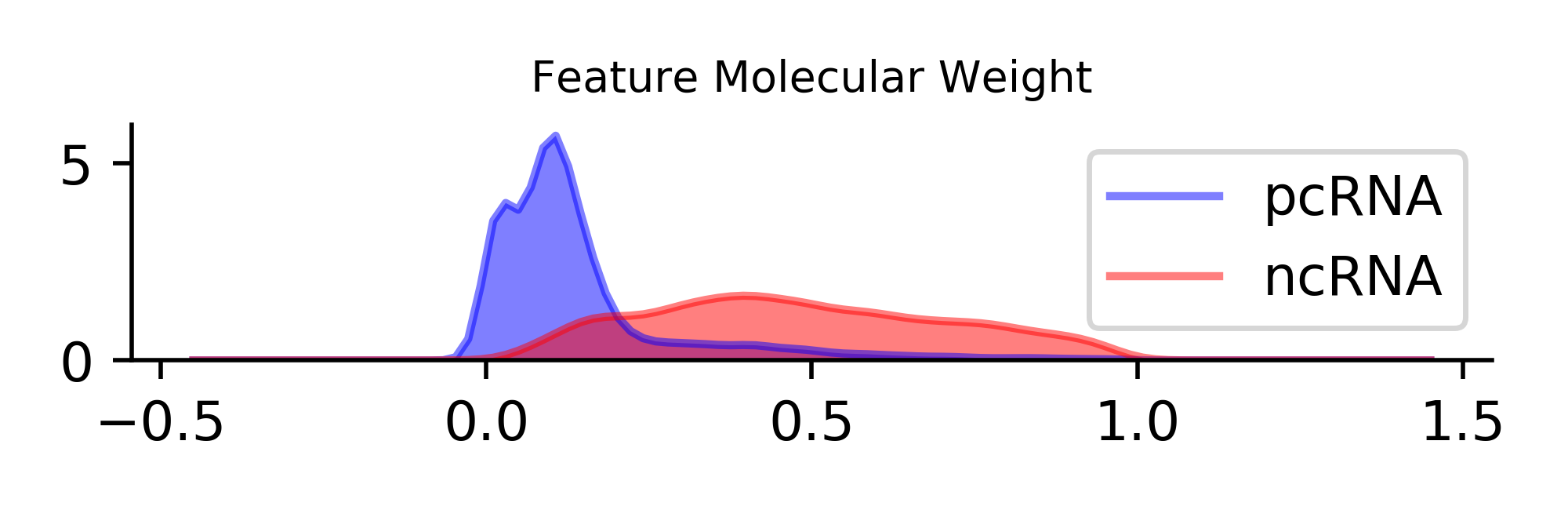 |
|  | 17 | Point Isoelectric | 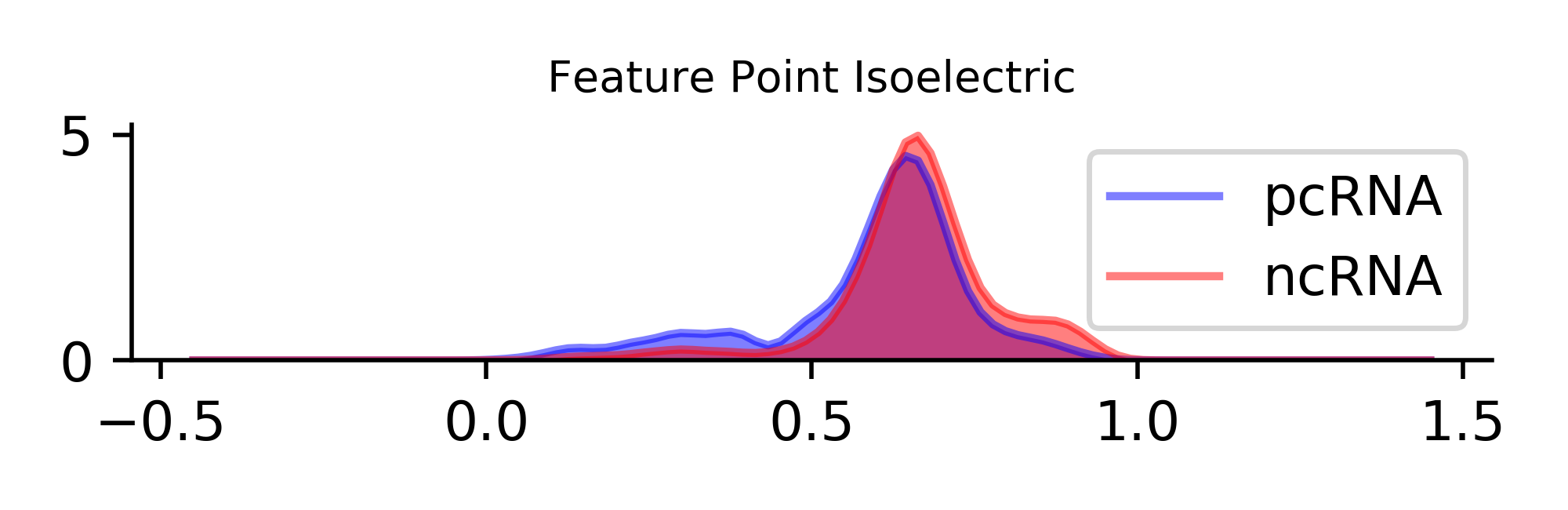 |
|  | 18 | Gravy | 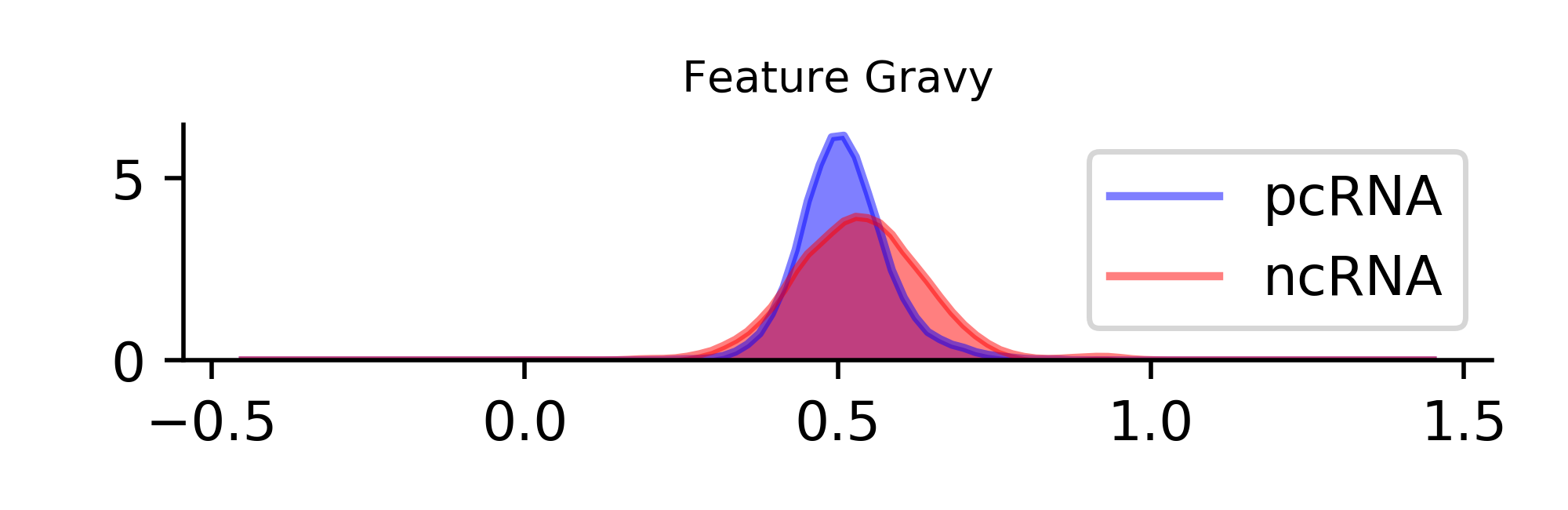 |
|  | 19 | Instability index | 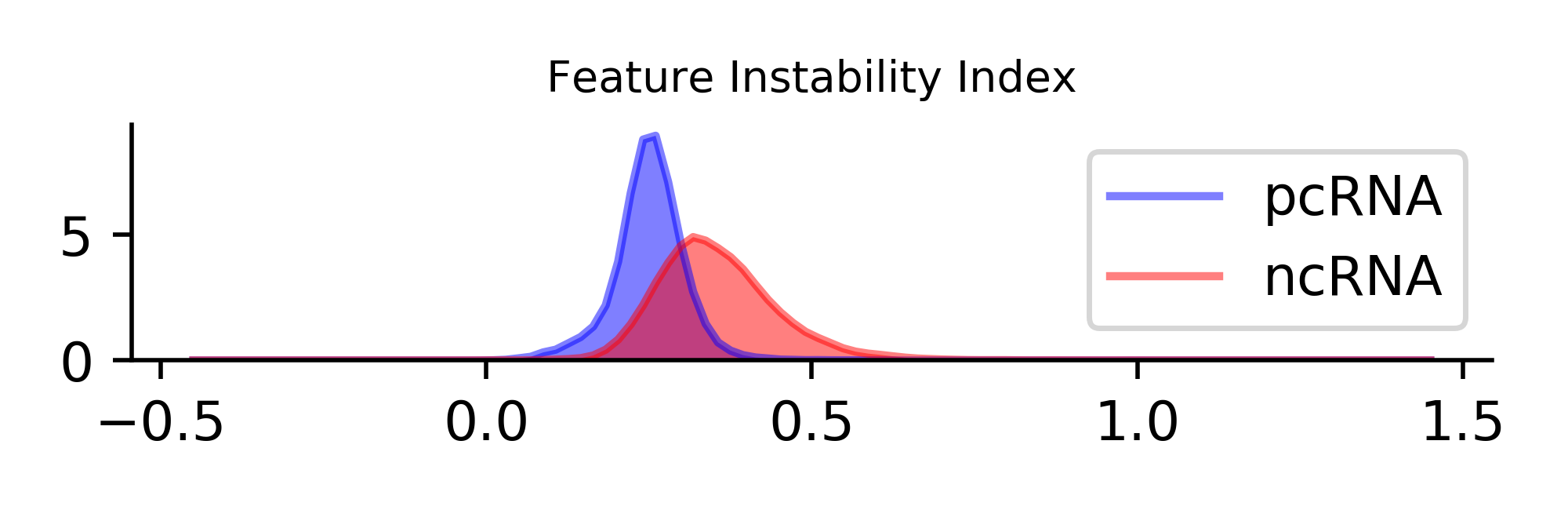 |
|  | 20 | Ratio of Point isoelectric and molecular weight | 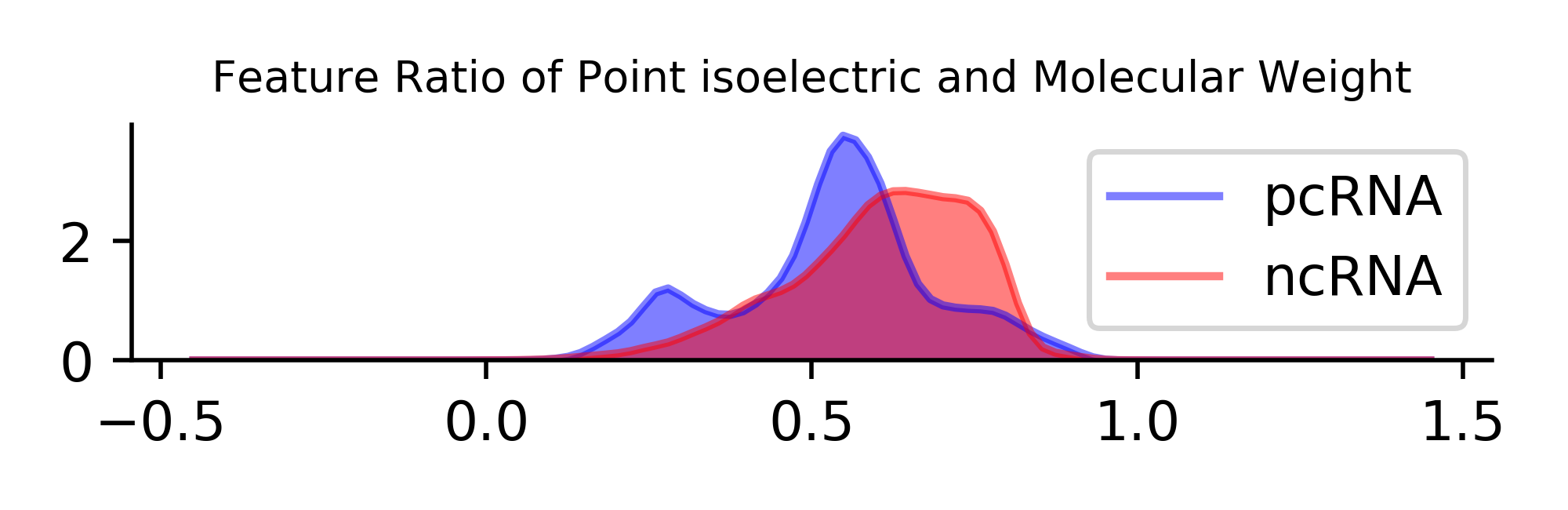 |
|  | 21 | Variance of ratio of Pont isoelectric and Molecular Weight | 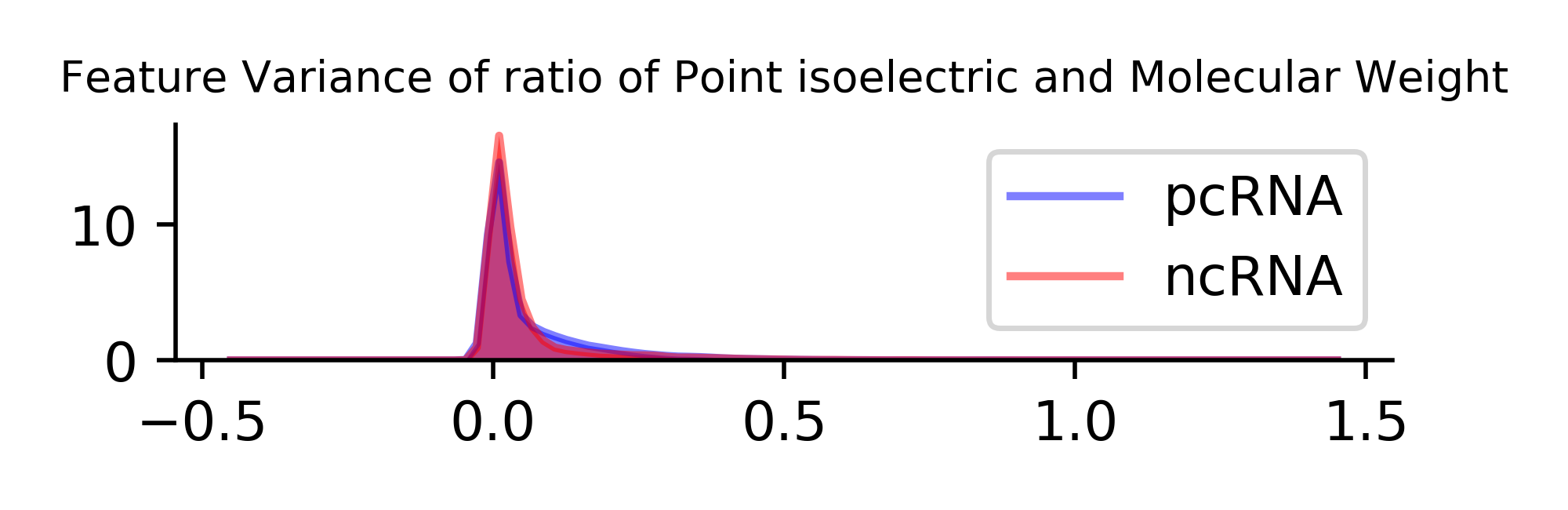 |
| RNA Physicochemical property features | 22 | Signal.Peak | 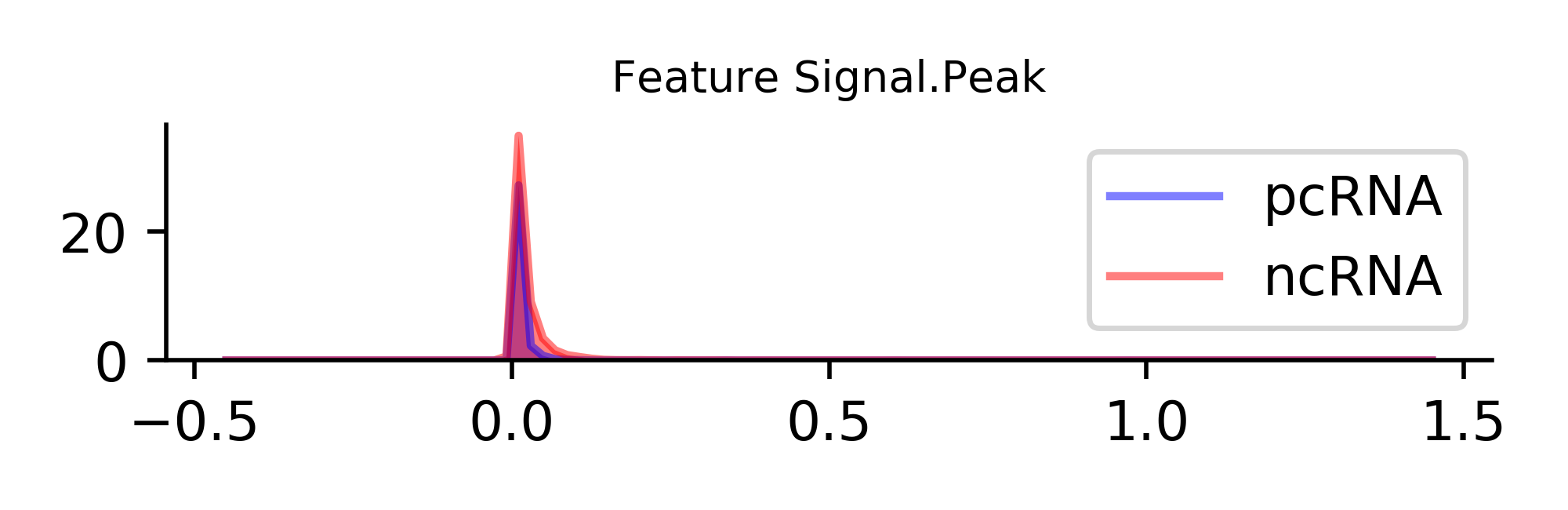 |
|  | 23 | SNR | 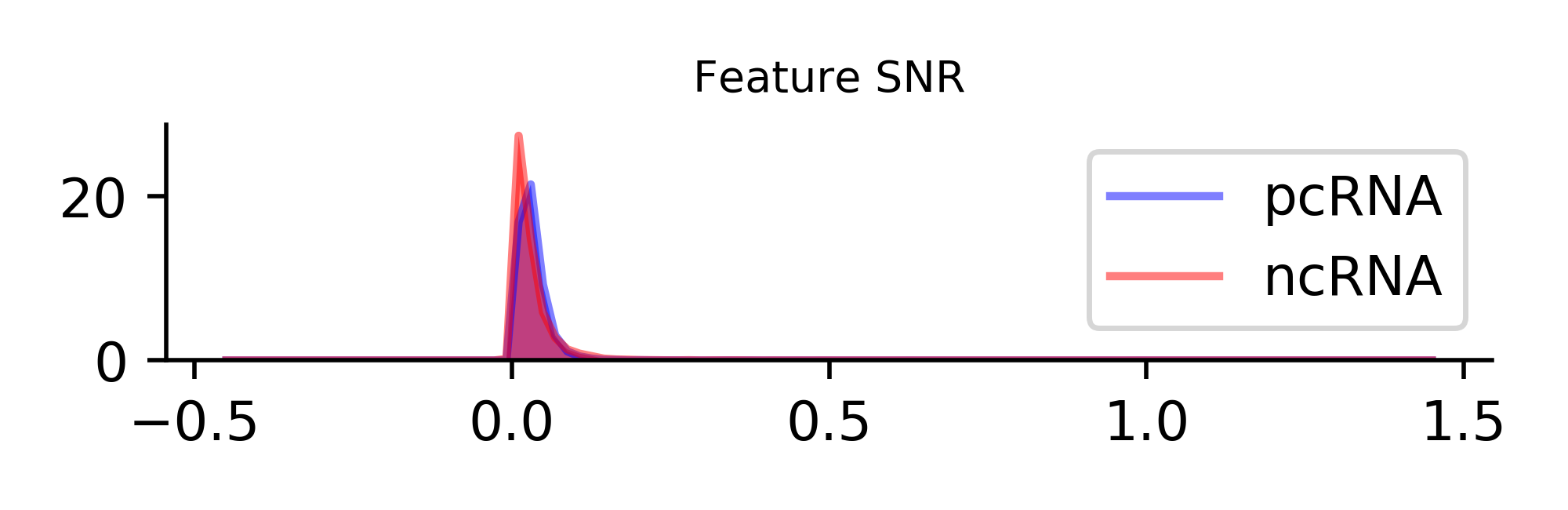 |
|  | 24 | Signal.Min | 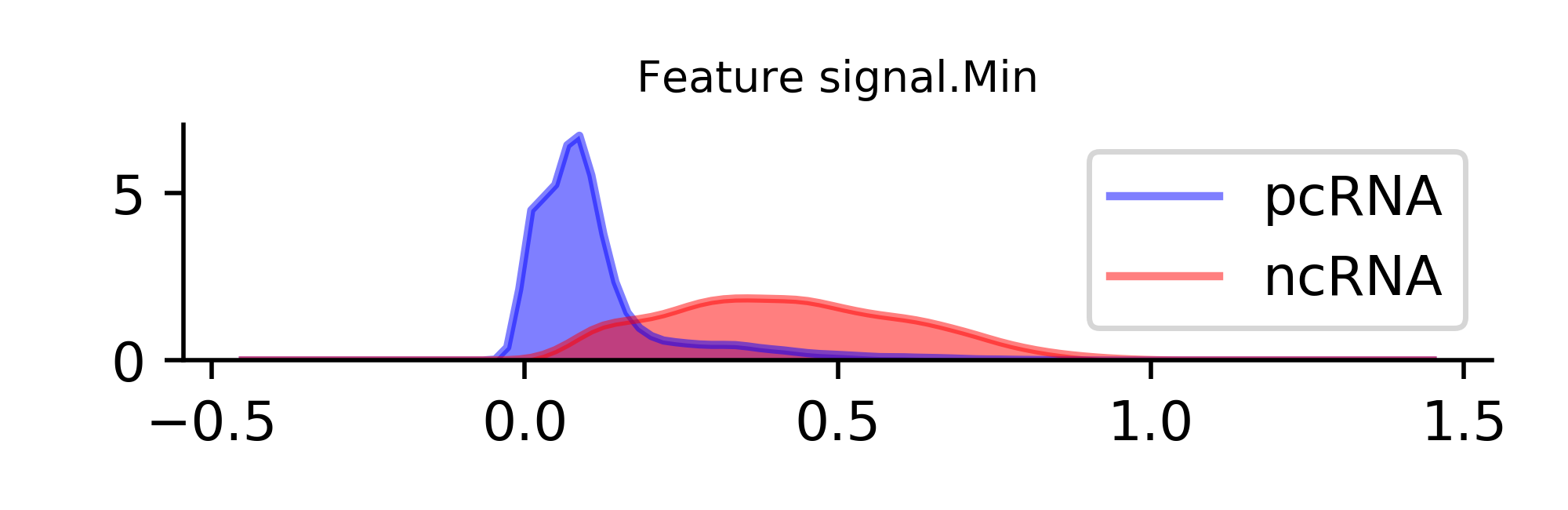 |
|  | 25 | Signal.Q1 | 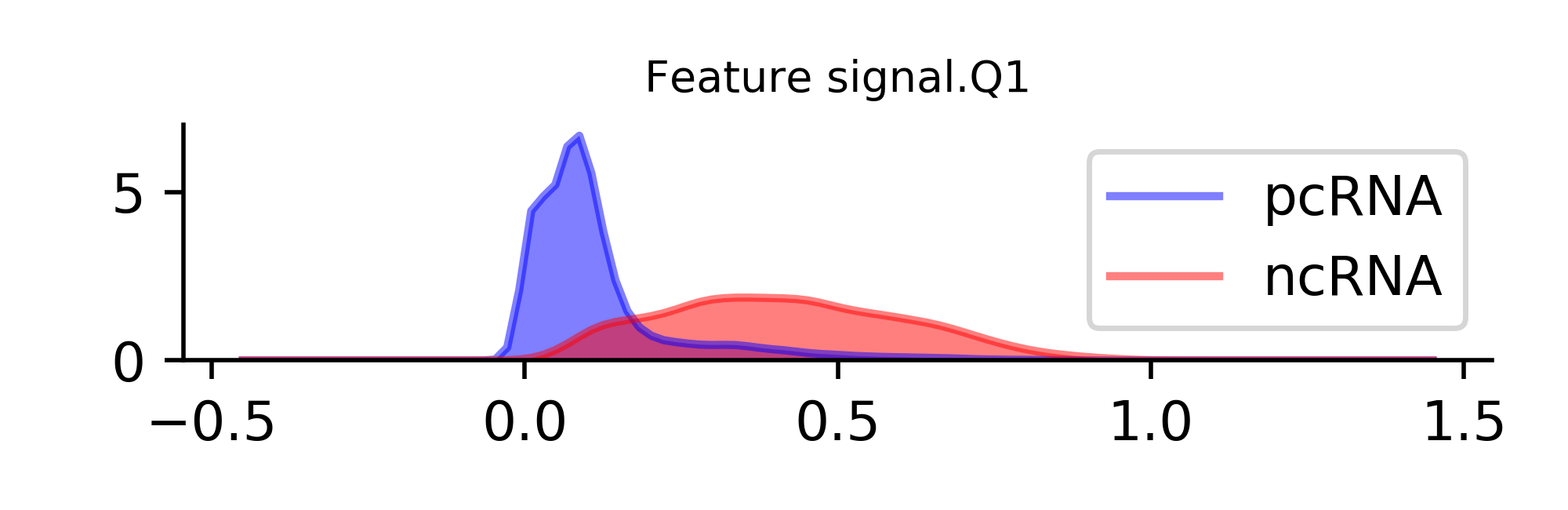 |
|  | 26 | Signal.Q2 | 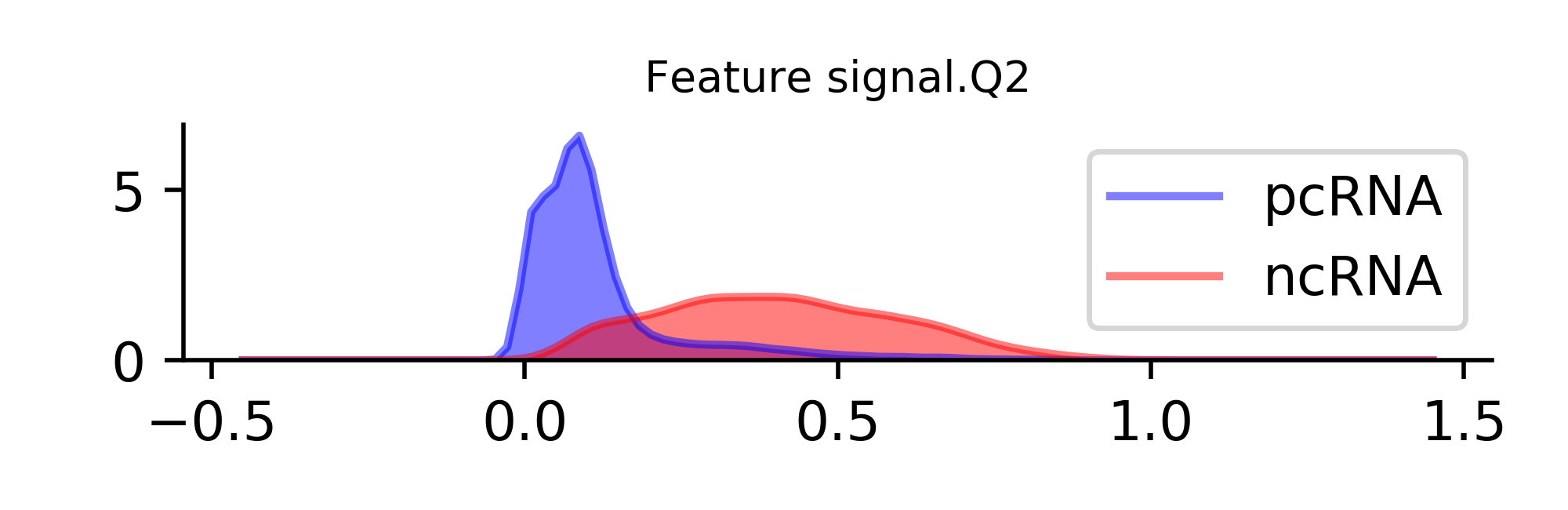 |
|  | 27 | Signal.Max | 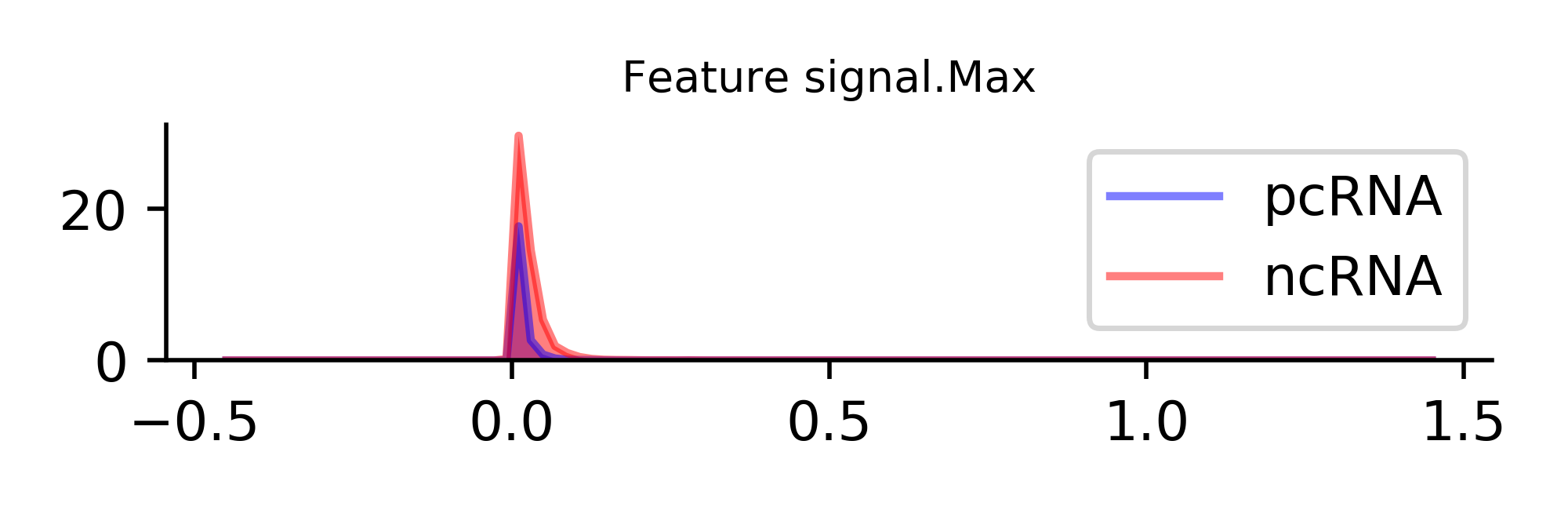 |
| Composition, Transition and Distribution features | 28 | A | 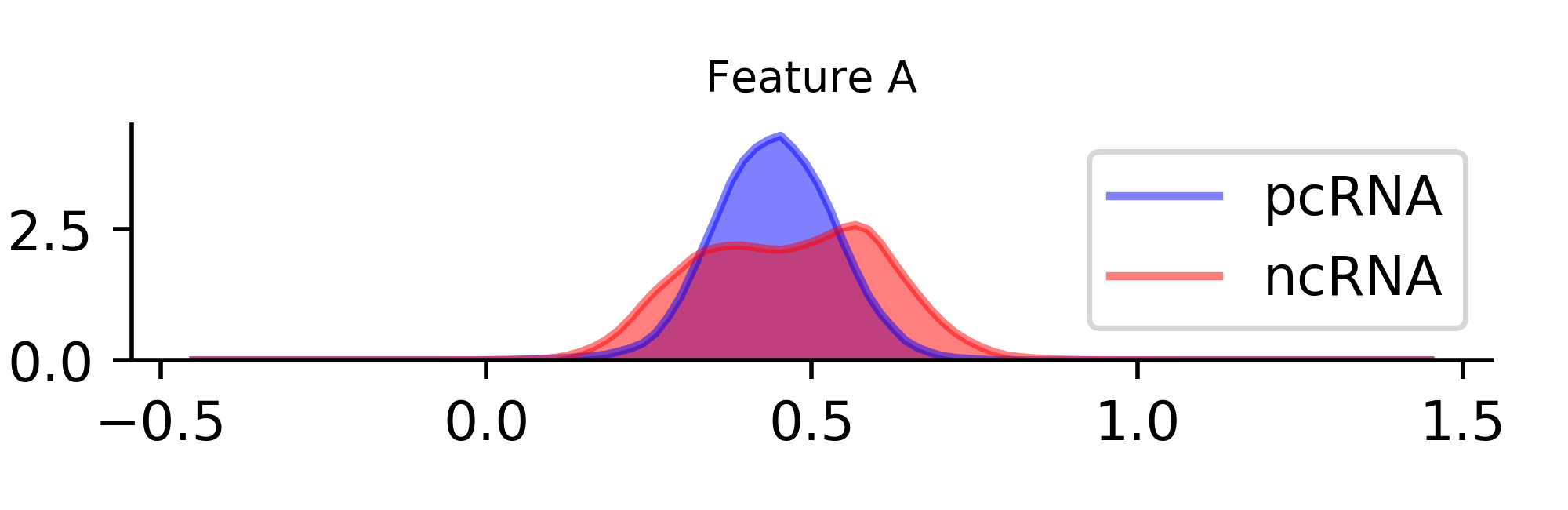 |
|  | 29 | T | 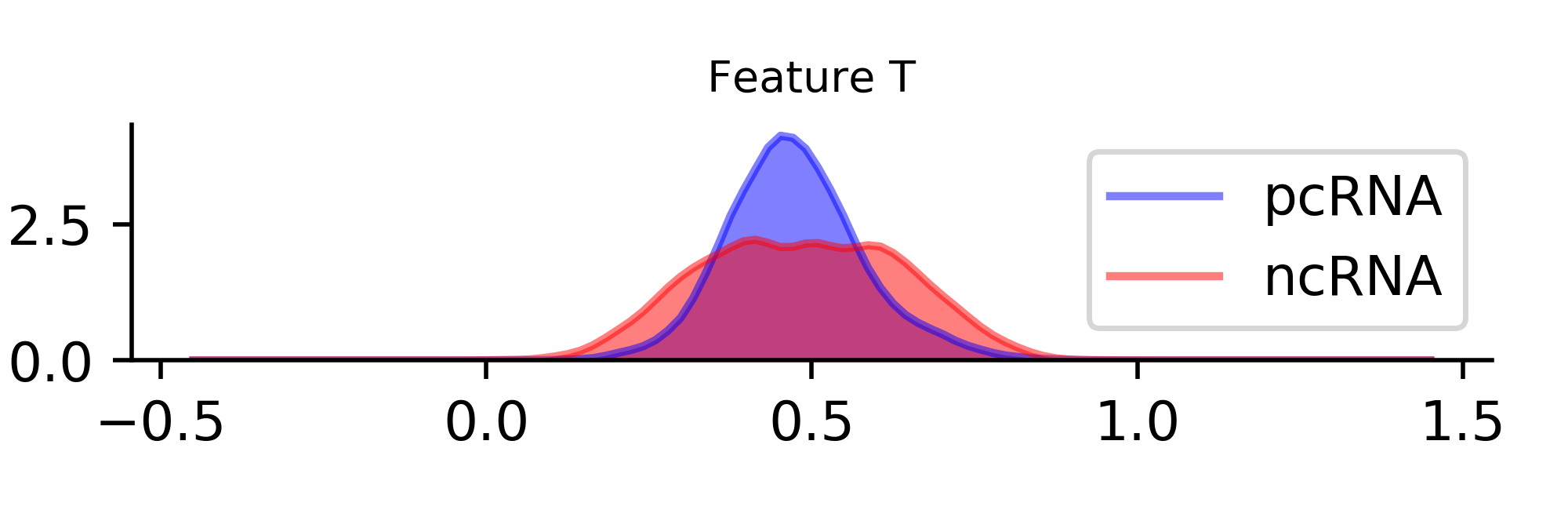 |
|  | 30 | G | 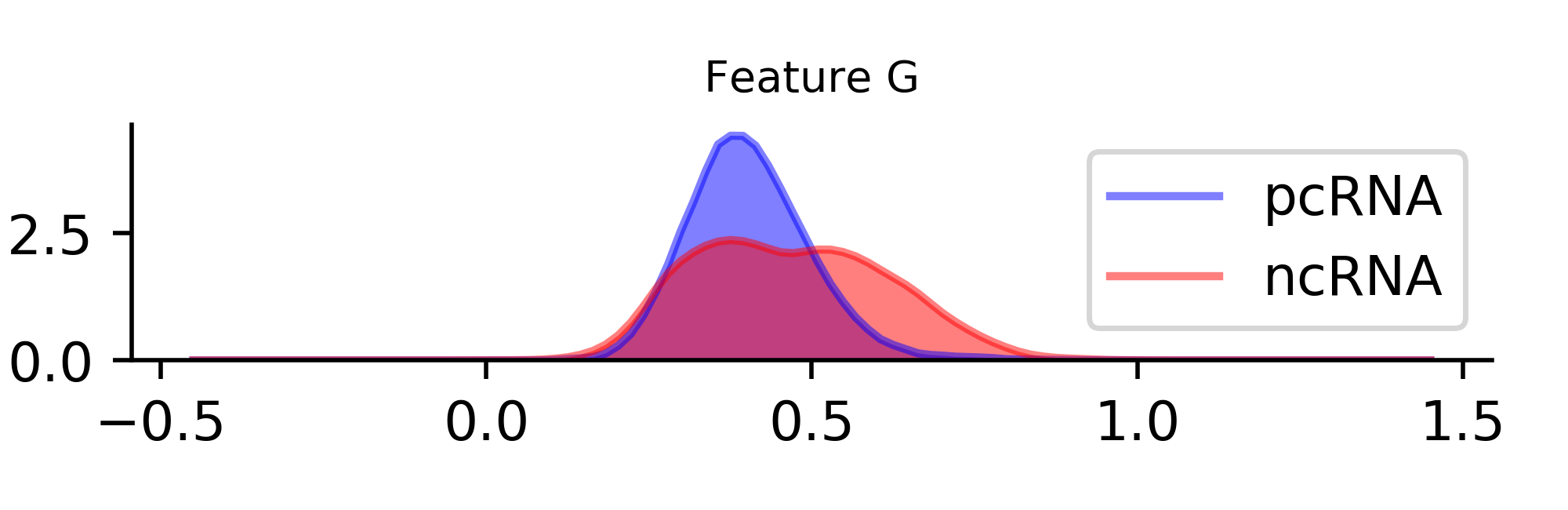 |
|  | 31 | C | 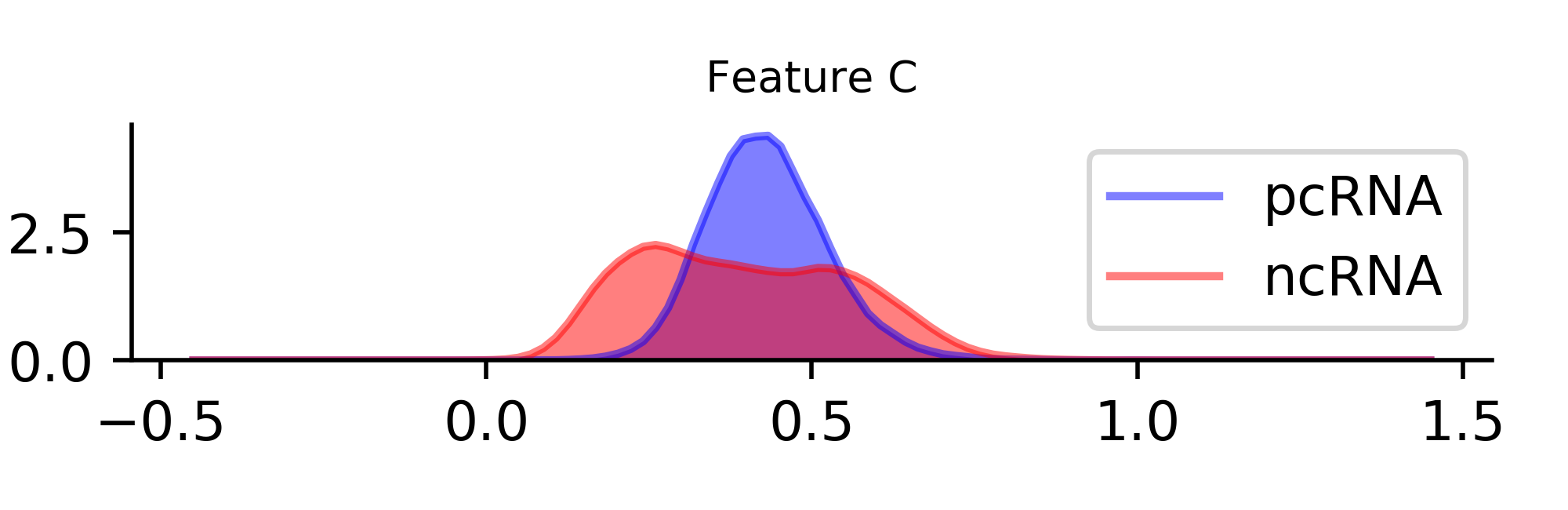 |
|  | 32 | AT | 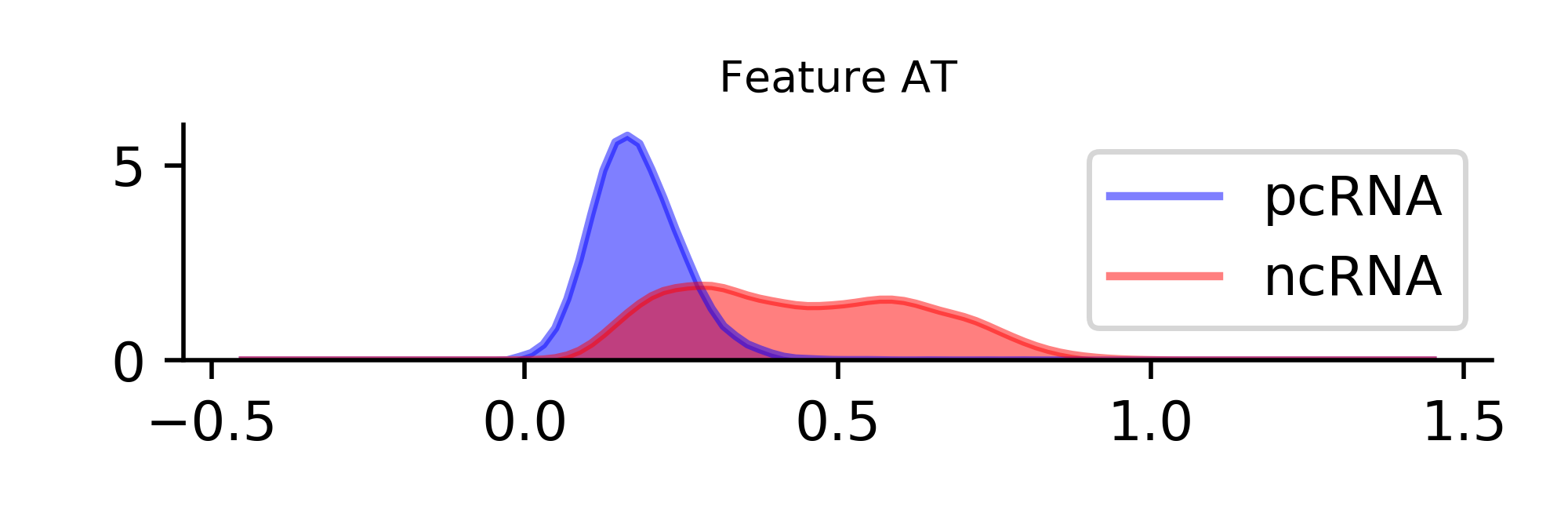 |
|  | 33 | AG | 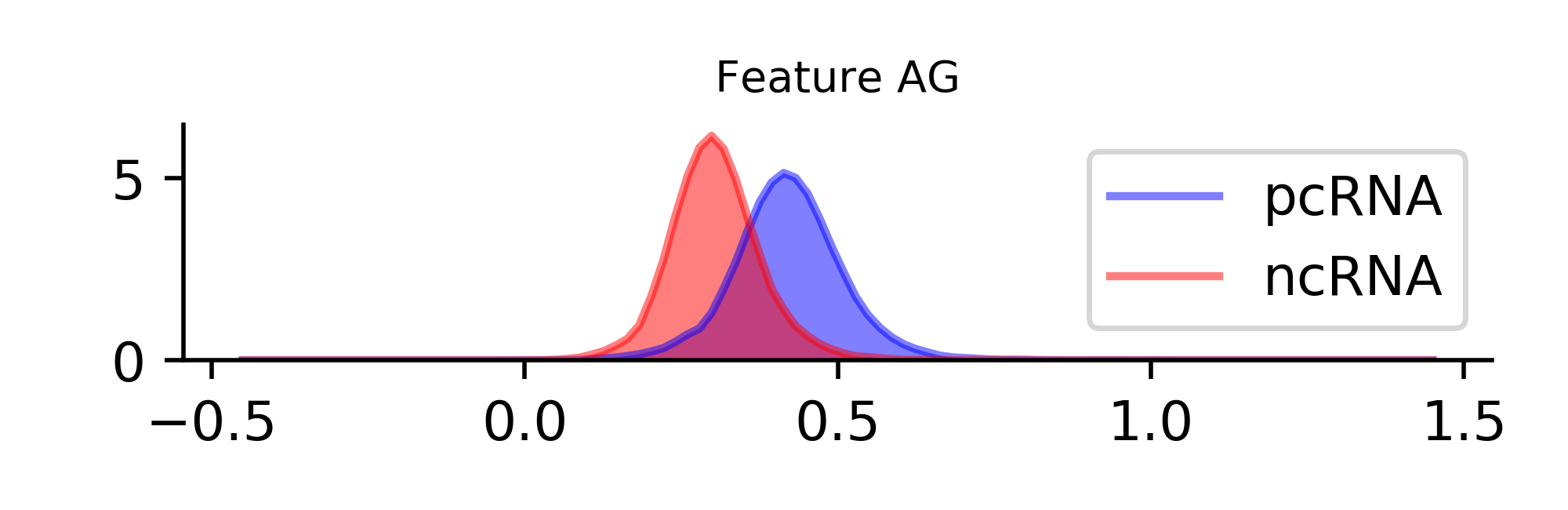 |
|  | 34 | AC | 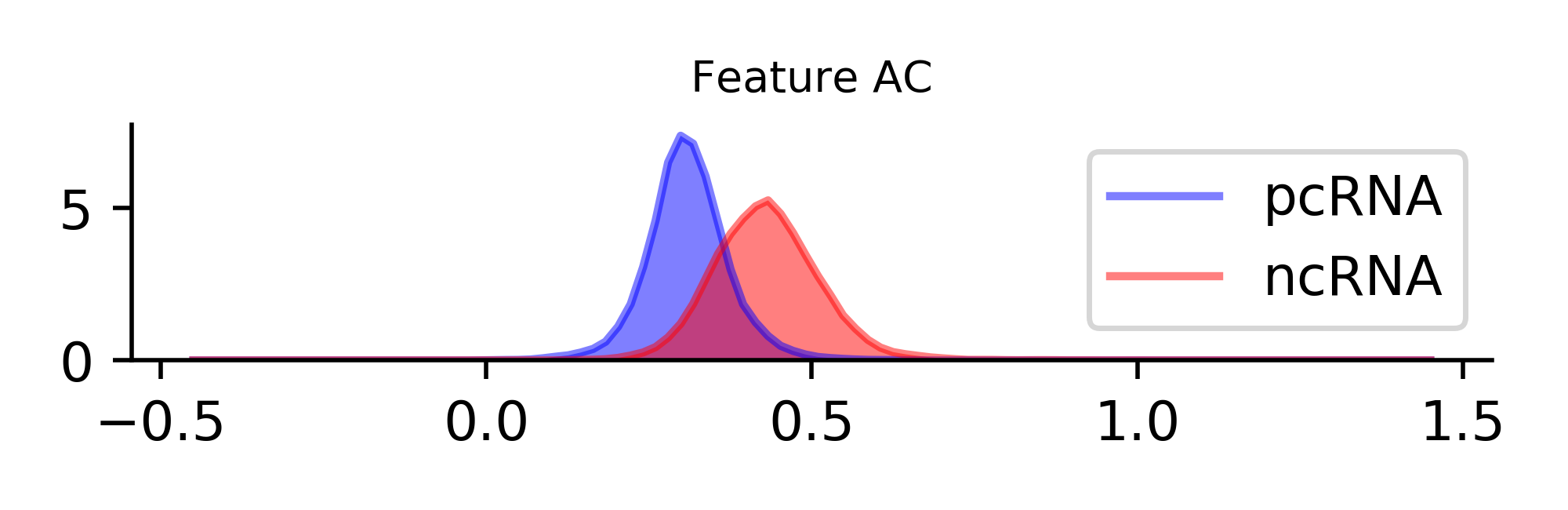 |
|  | 35 | TG | 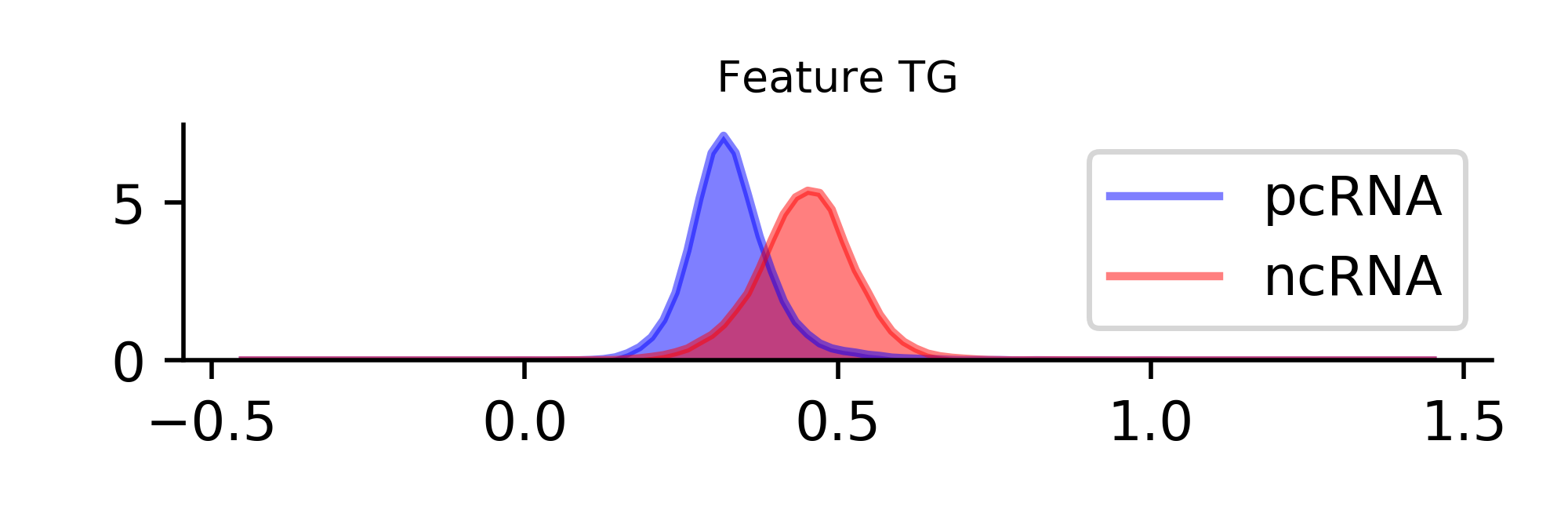 |
|  | 36 | TC | 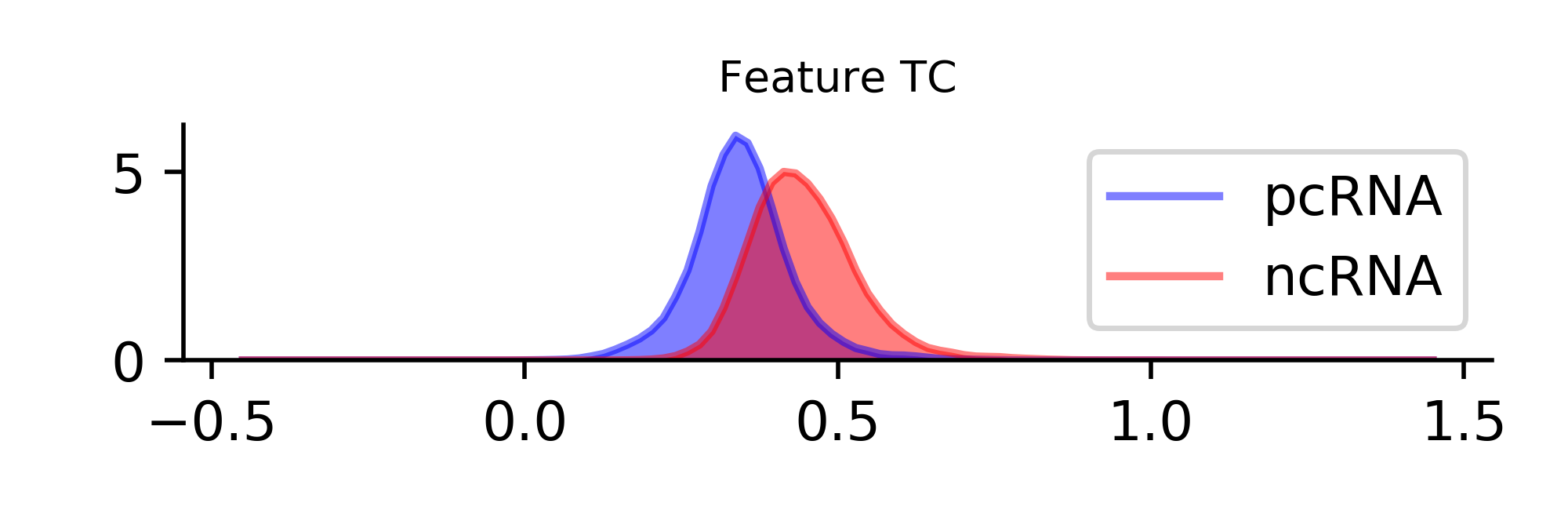 |
|  | 37 | GC | 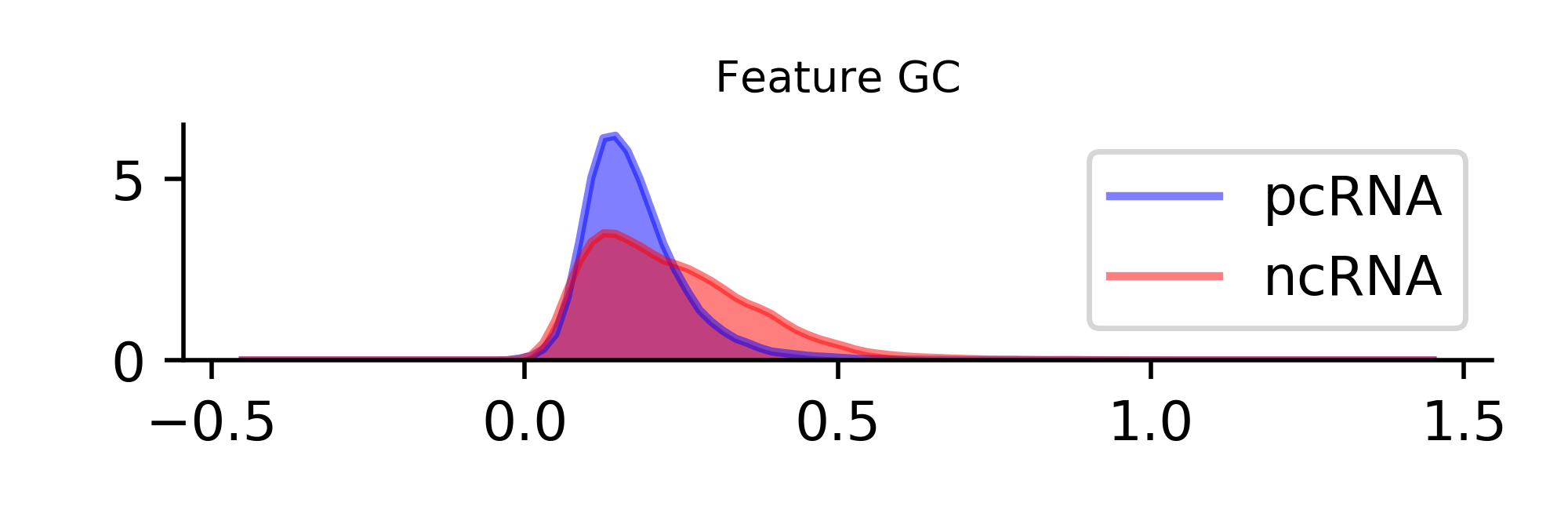 |
|  | 38 | A0 | 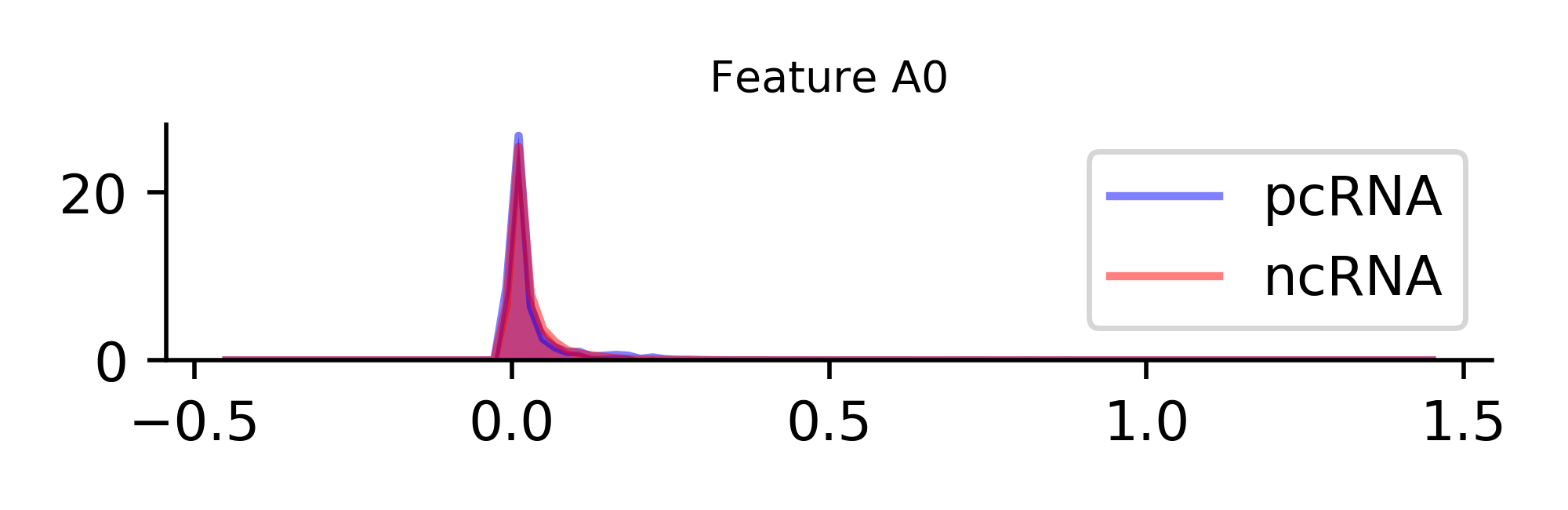 |
|  | 39 | A1 | 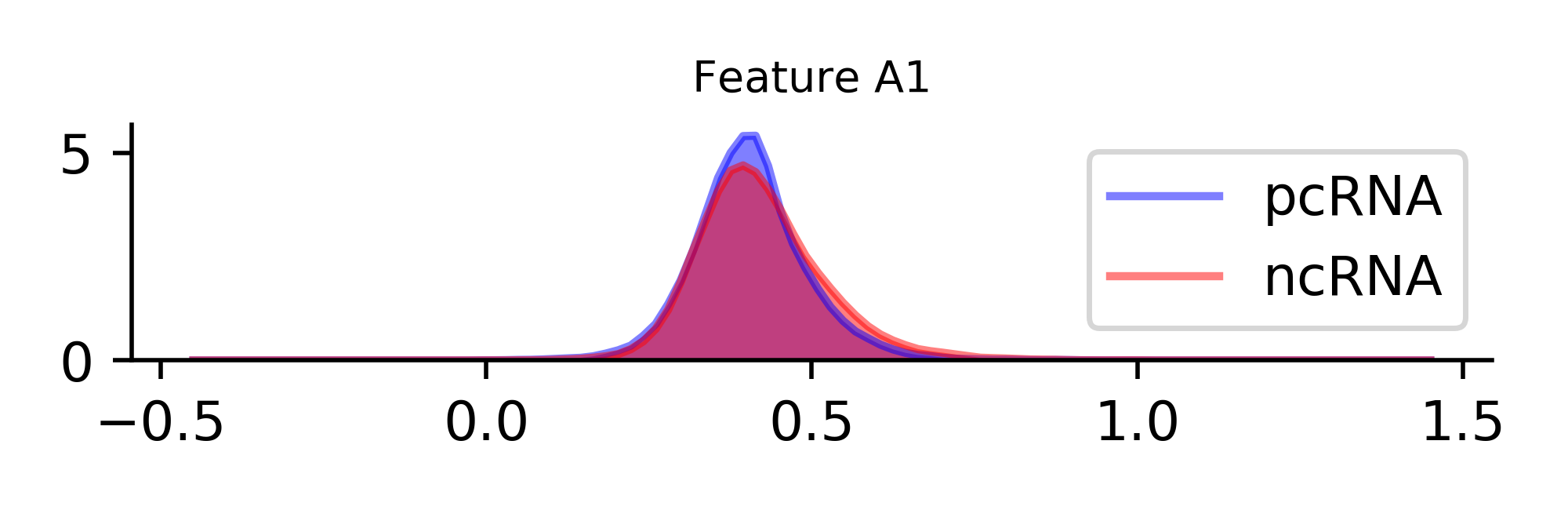 |
|  | 40 | A2 | 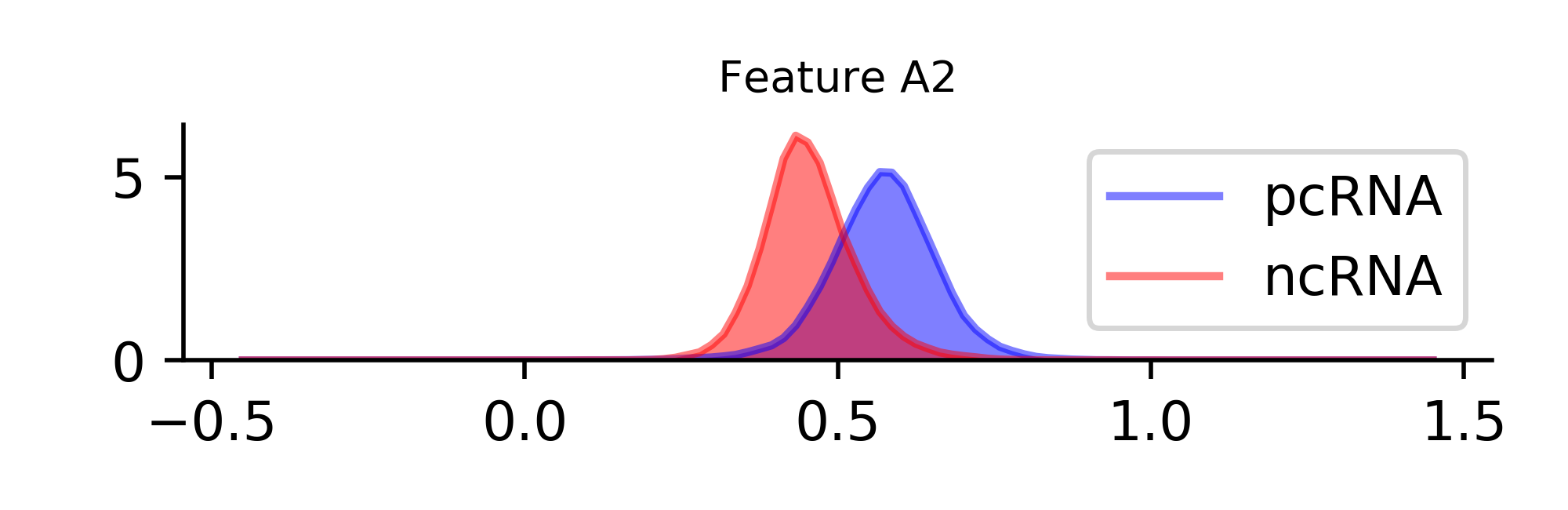 |
|  | 41 | A3 | 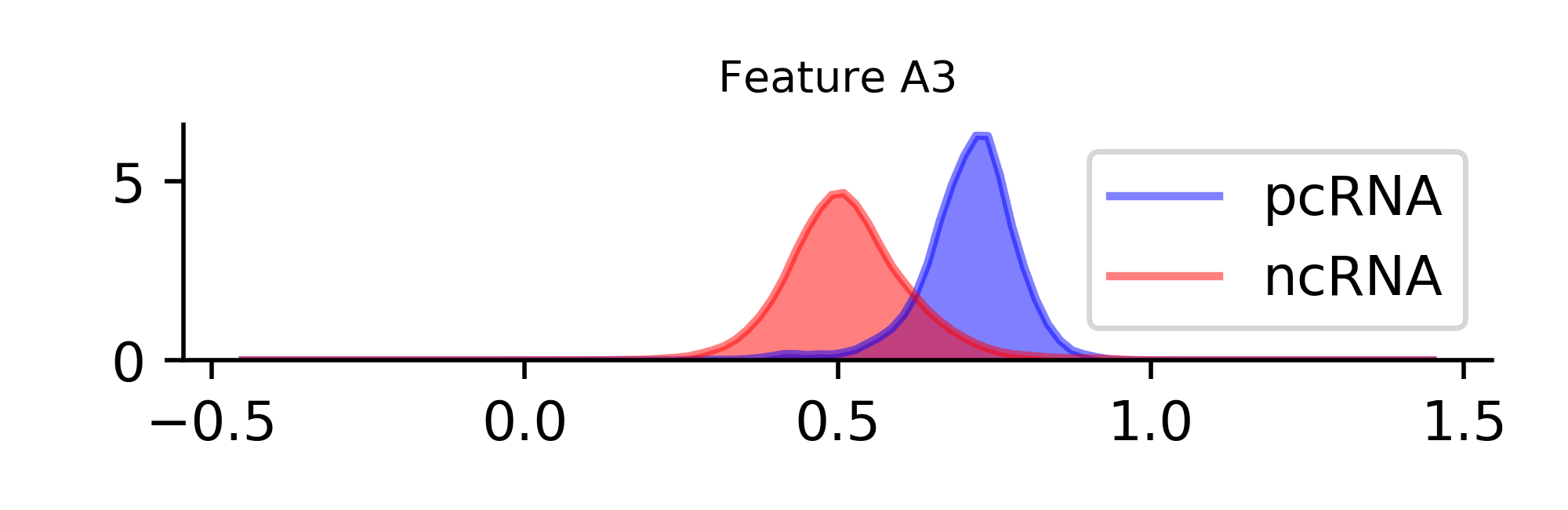 |
|  | 42 | A4 | 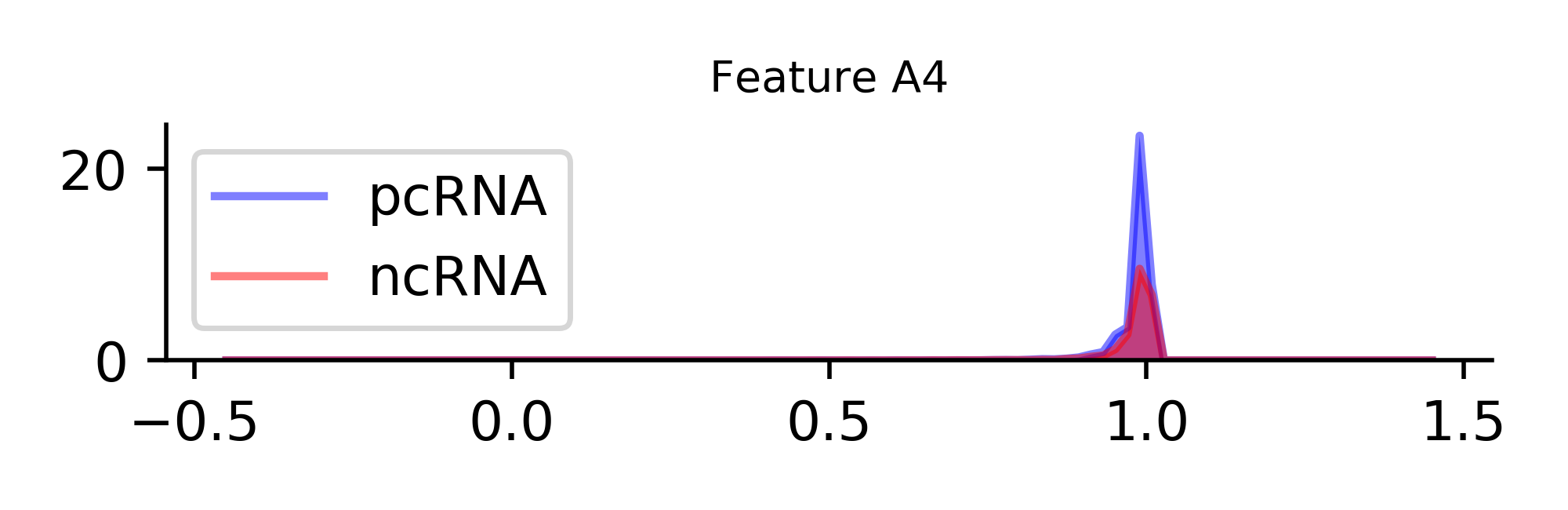 |
|  | 43 | T0 | 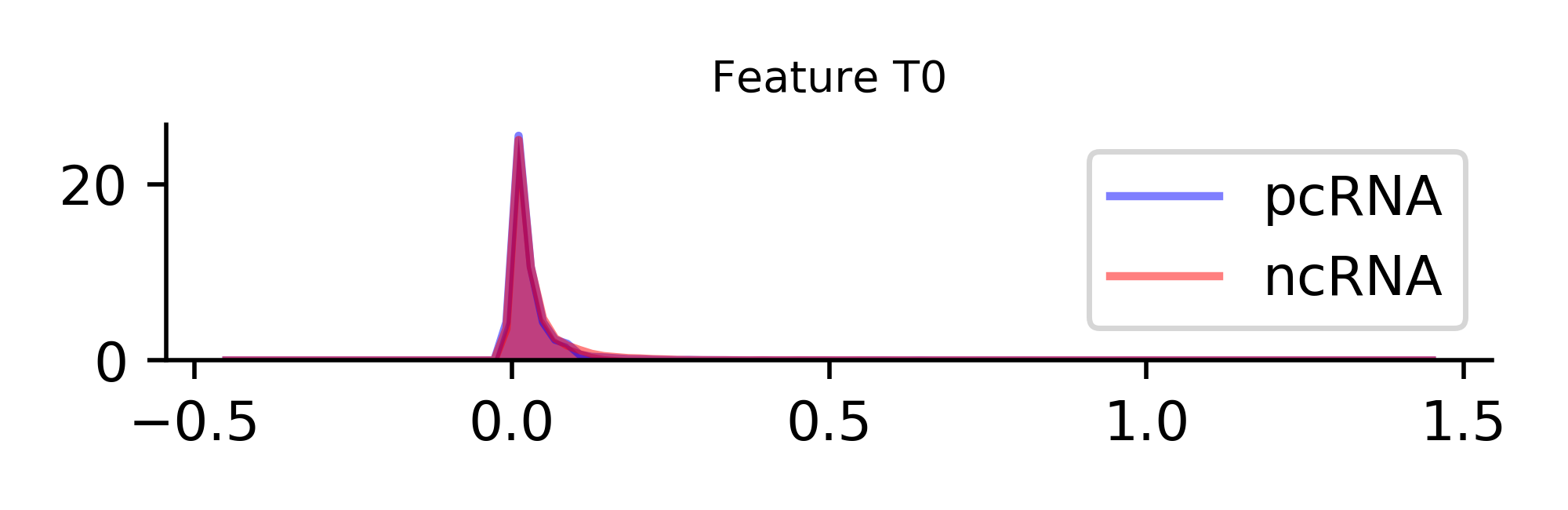 |
|  | 44 | T1 | 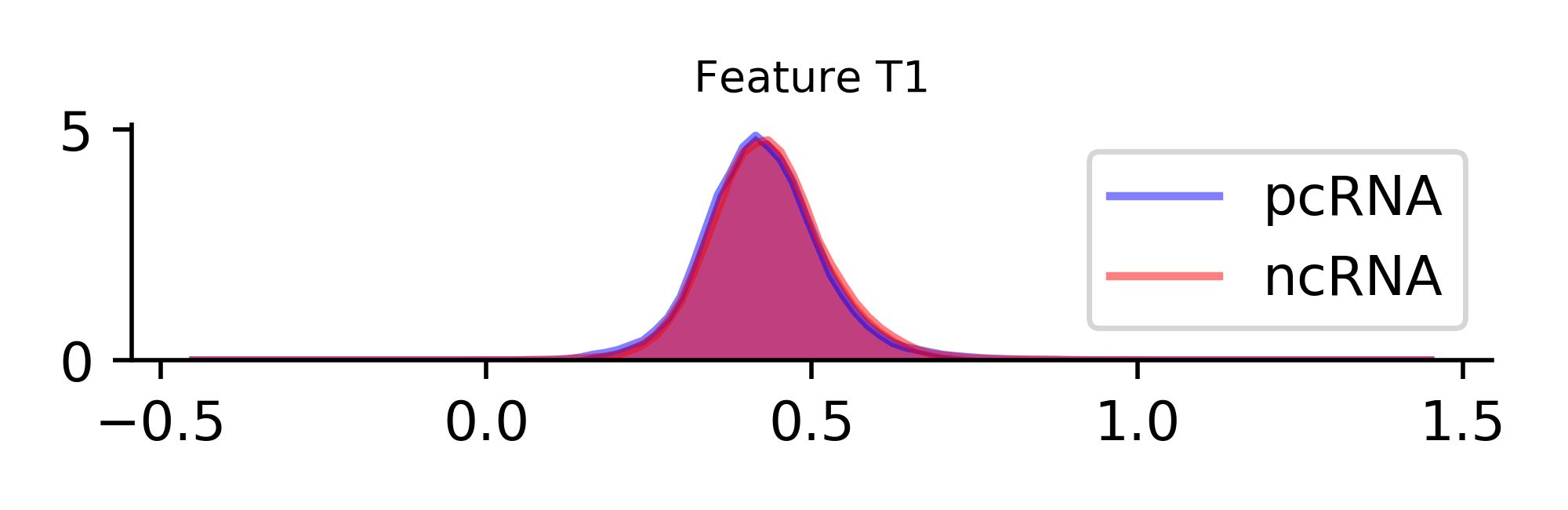 |
|  | 45 | T2 | 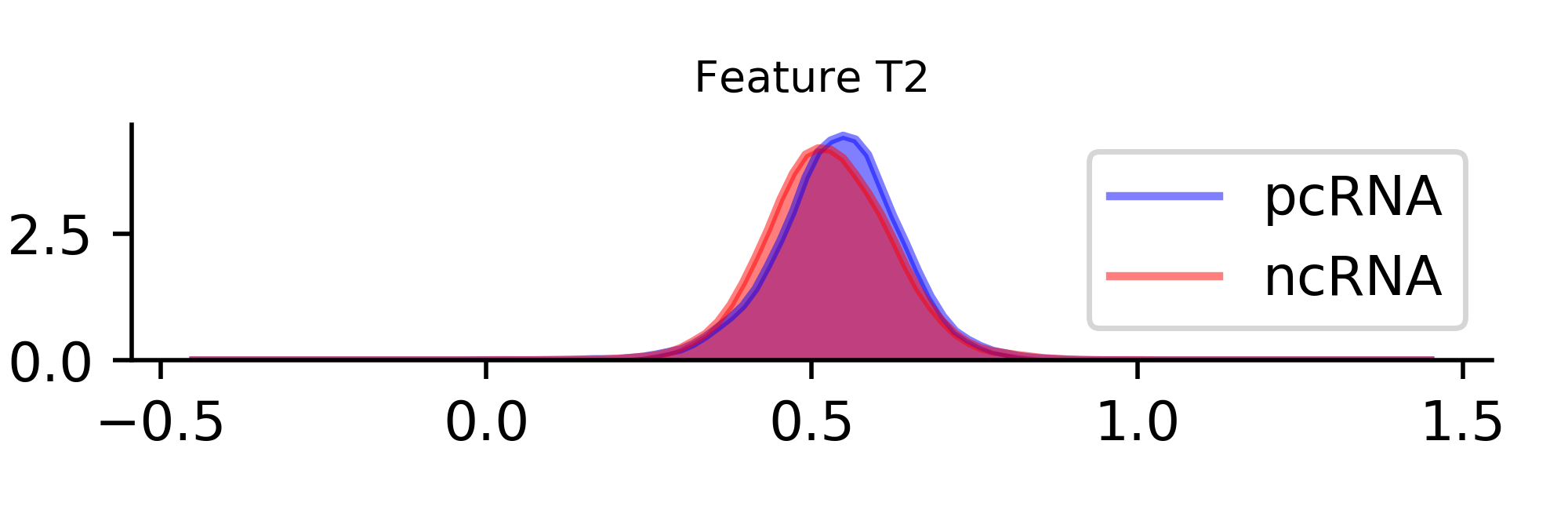 |
|  | 45 | T3 | 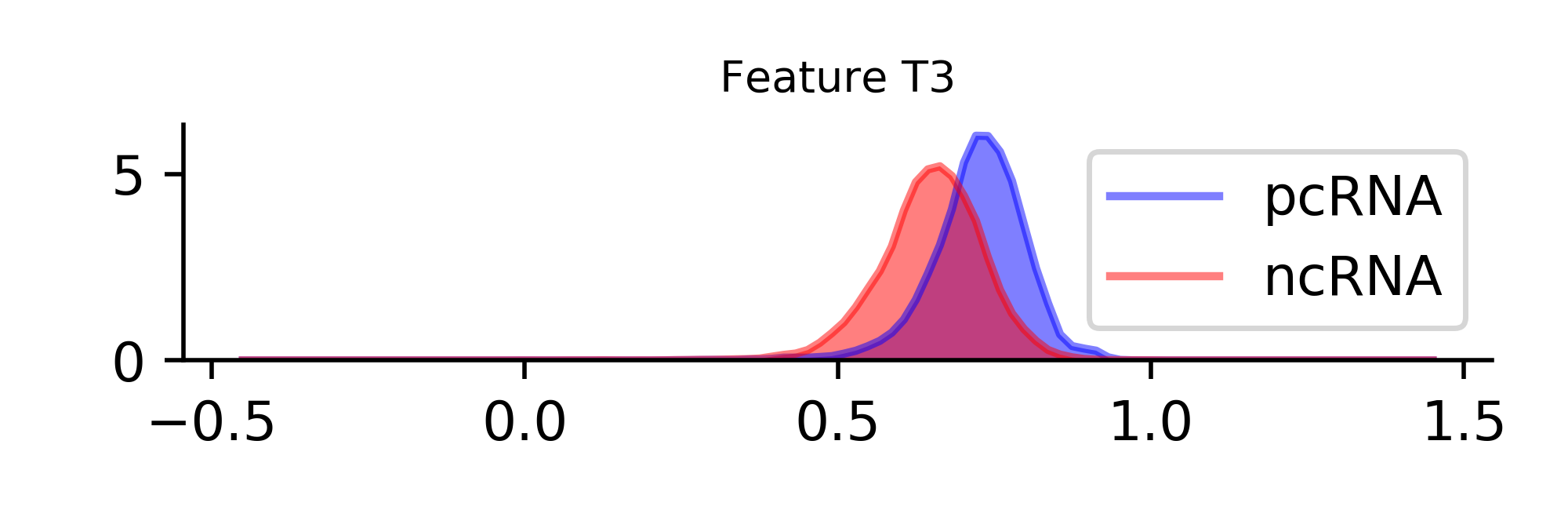 |
|  | 47 | T4 | 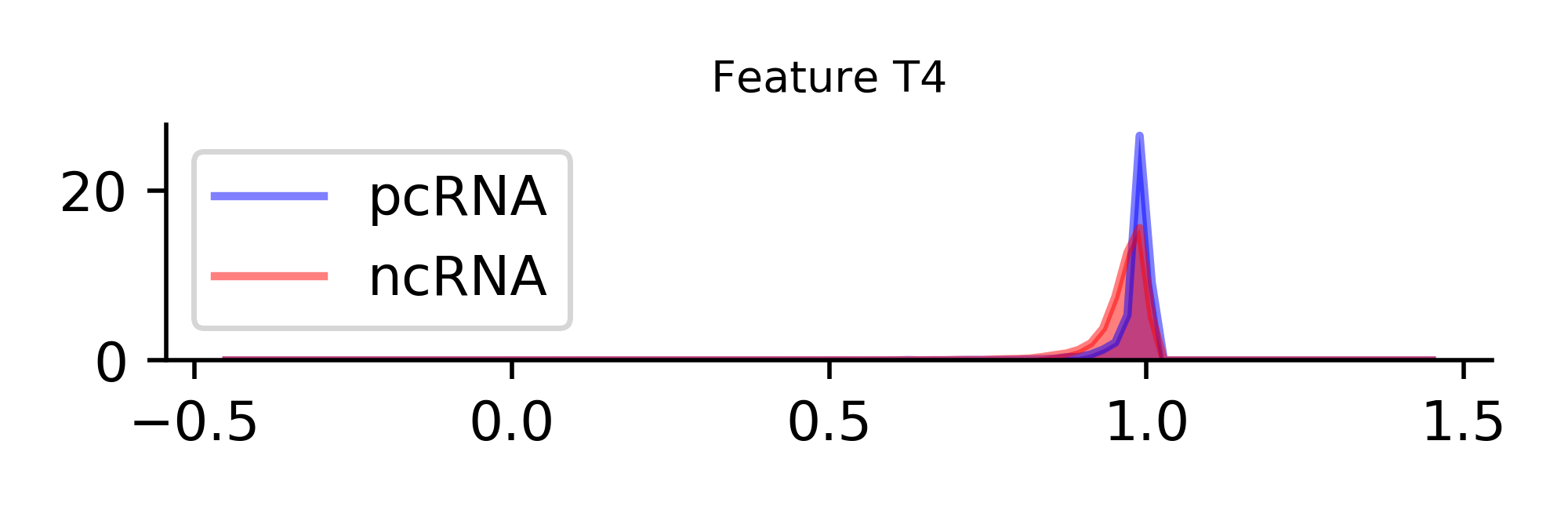 |
|  | 48 | C0 | 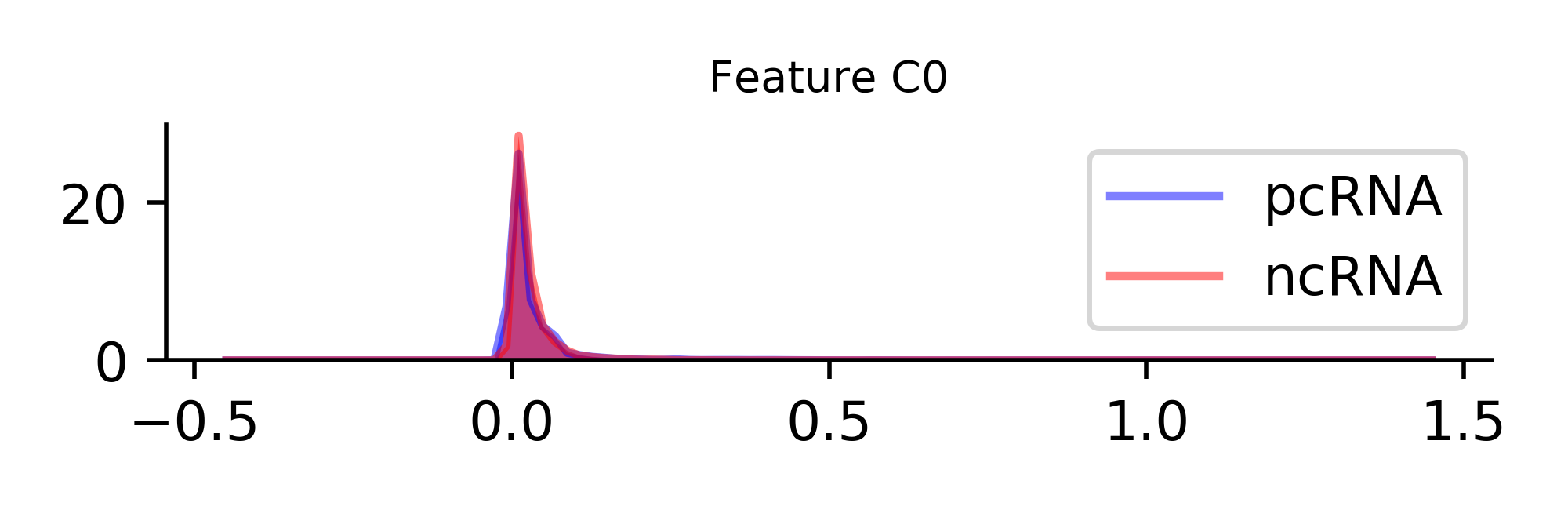 |
|  | 49 | C1 | 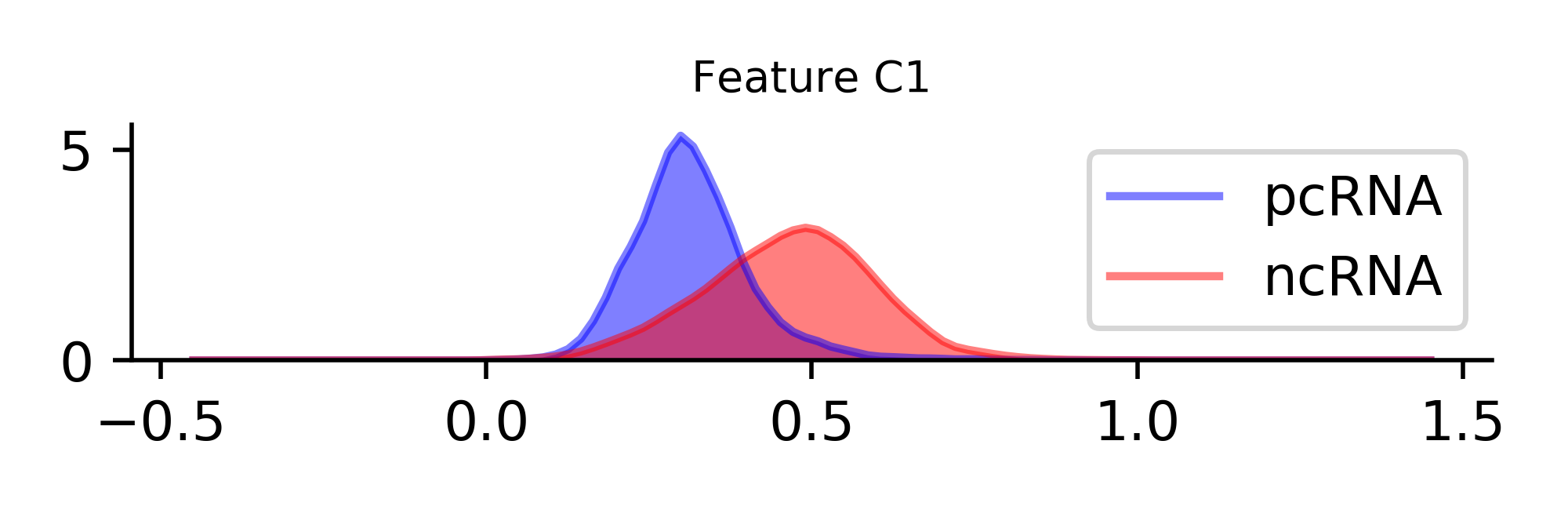 |
|  | 50 | C2 | 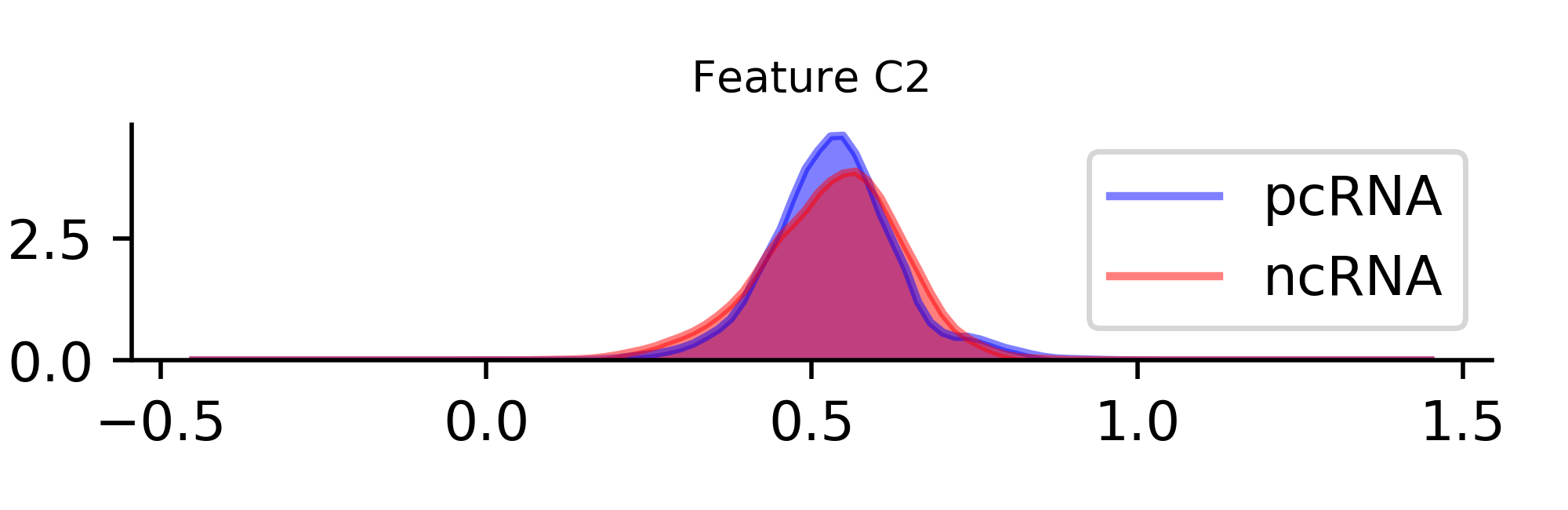 |
|  | 51 | C3 | 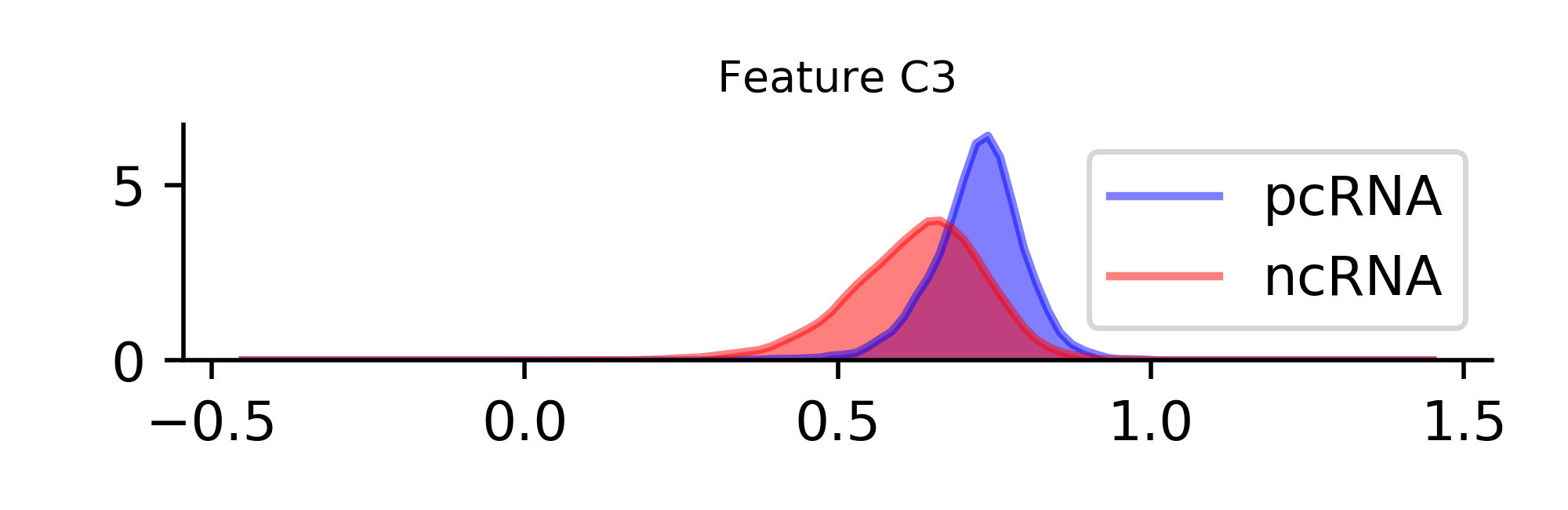 |
|  | 52 | C4 |  |
|  | 53 | G0 | 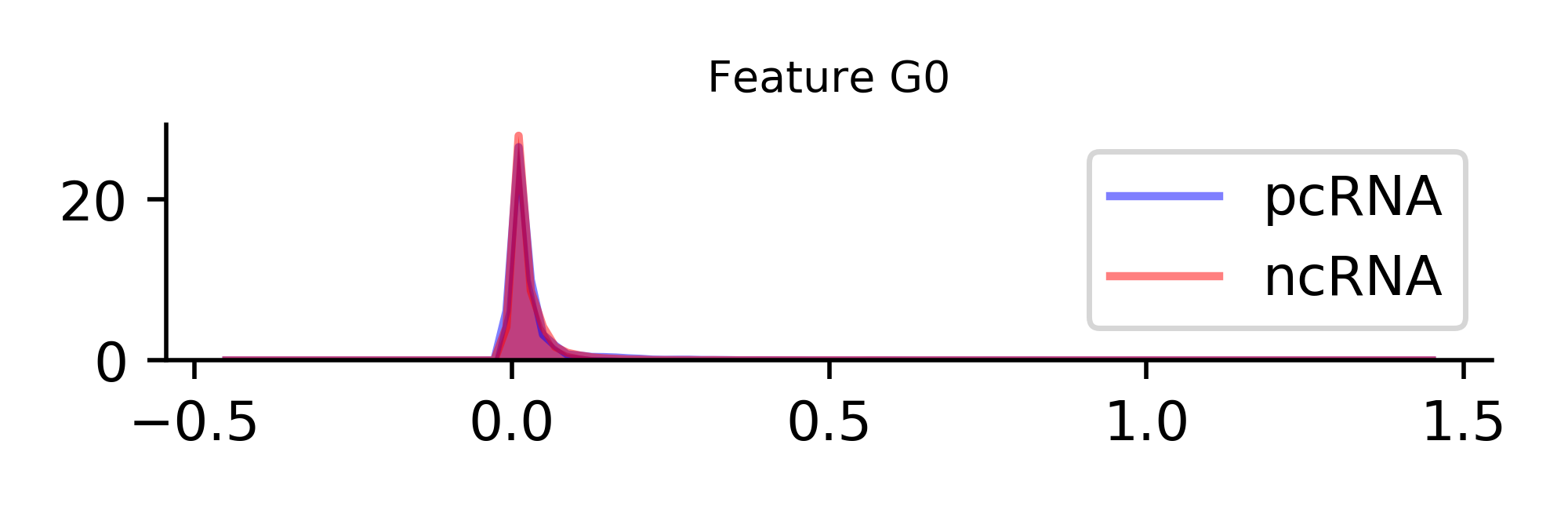 |
|  | 54 | G1 | 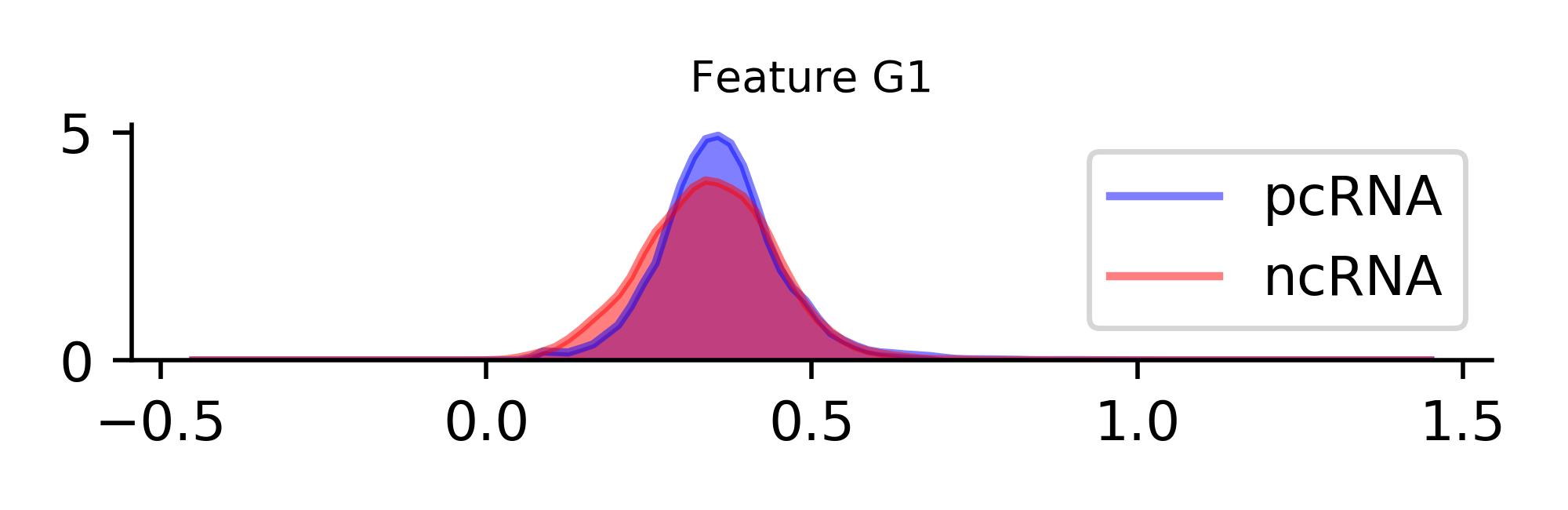 |
|  | 55 | G2 | 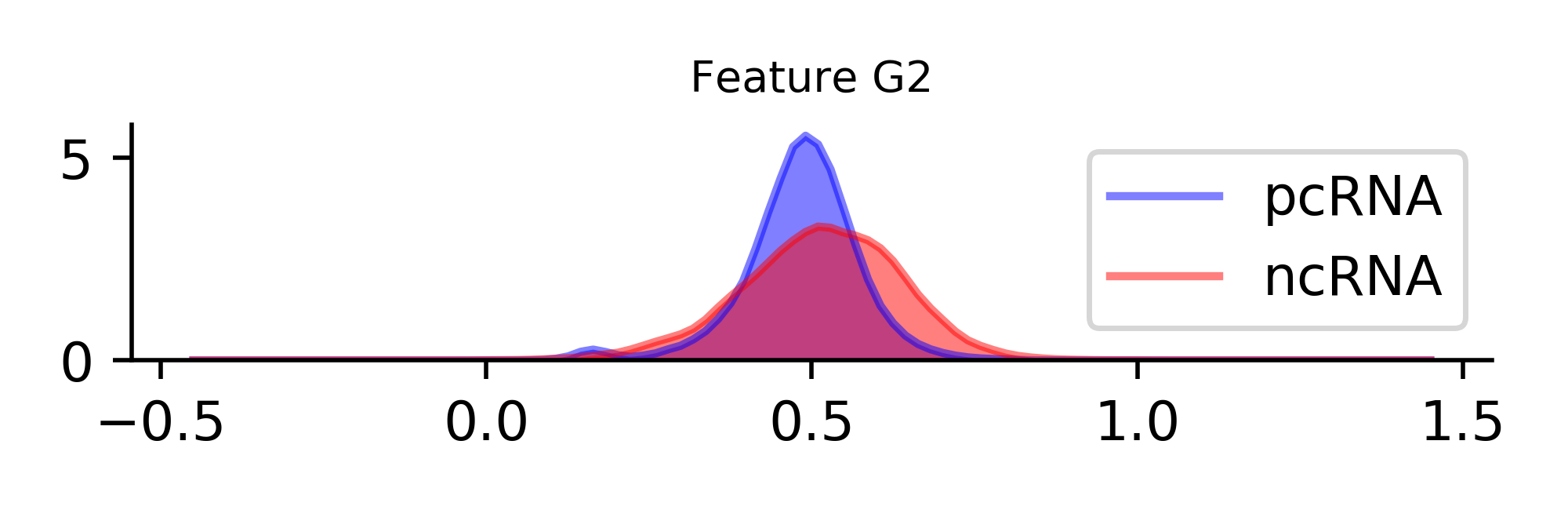 |
|  | 56 | G3 | 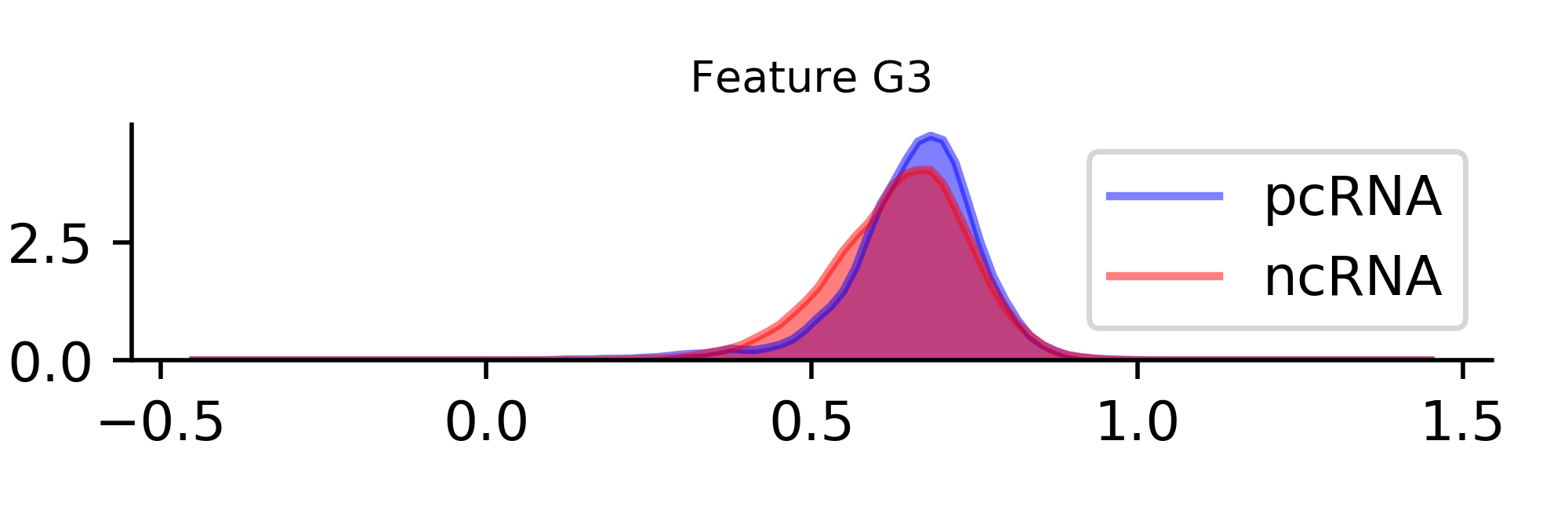 |
|  | 57 | G4 | 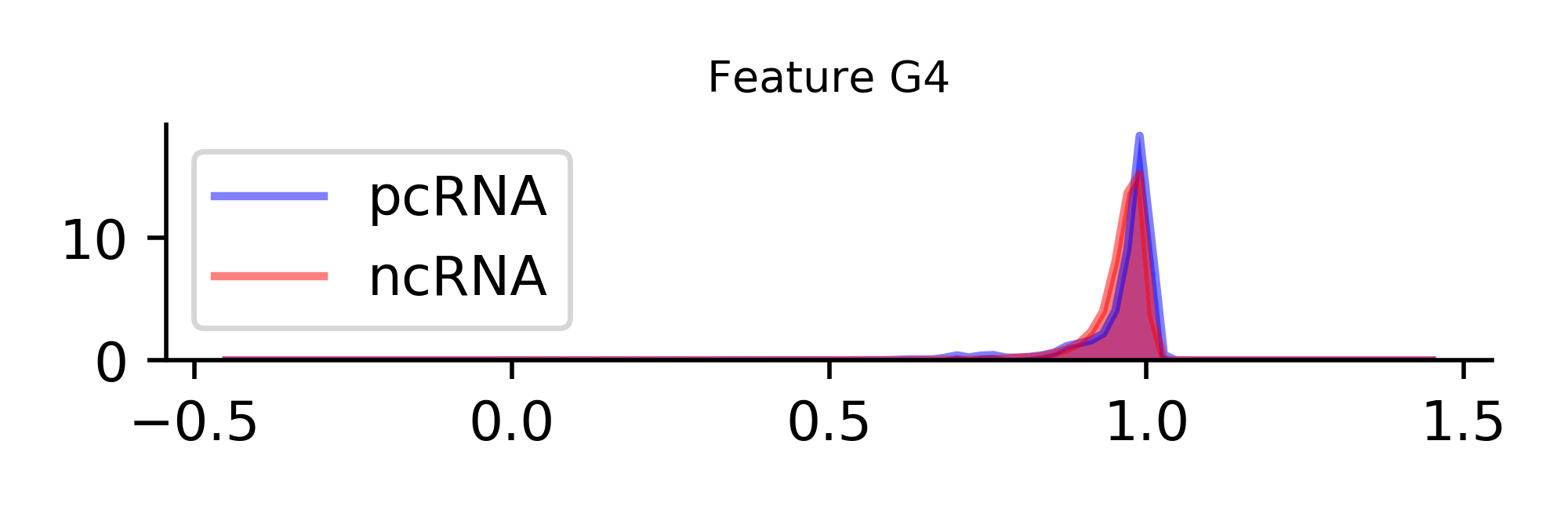 |

**The user's manual of NCResNet**

1. **Data and source code are available**

Data and source code are available at <https://github.com/abcair/NCResNet>. Anyone can download or clone the data and source code freely.

1. **How to use NCResNet**
   1. Create a Python=3.5 virtual environments by Conda
   2. Install dependency package
   3. Run NCResNet
2. **There is easy demo how to run NCResNet**
3. Create running environment
   - 1. conda create -n NCResNetEnv python=3.5
     2. conda activate NCResNetEnv
4. Install dependency package
5. conda install r
6. install.packages("LncFinder")
7. pip install numpy
8. pip install pandas
9. pip install sklearn
10. pip install biopython
11. pip install tensorflow
12. pip install keras
13. pip install rpy2==3.0.1
14. Run NCResNet

NCResNet receives fasta format RNA sequence as input and output a tsv format result file

python test.py -i ./demo.fasta -m NCResNet.h5 -o result.tsv
